# *Toxoplasma gondii* Genotyping: A Closer Look Into Europe

**DOI:** 10.3389/fcimb.2022.842595

**Published:** 2022-03-23

**Authors:** Mercedes Fernández-Escobar, Gereon Schares, Pavlo Maksimov, Maike Joeres, Luis Miguel Ortega-Mora, Rafael Calero-Bernal

**Affiliations:** ^1^SALUVET, Animal Health Department, Faculty of Veterinary Sciences, Complutense University of Madrid, Madrid, Spain; ^2^National Reference Laboratory for Toxoplasmosis, Institute of Epidemiology, Friedrich-Loeffler-Institut, Federal Research Institute for Animal Health, Greifswald-Insel Riems, Germany

**Keywords:** *Toxoplasma gondii*, Europe, genotypes, typing methodologies, population structure

## Abstract

*Toxoplasma gondii* is a major zoonotic agent which may cause harmful effects mainly in pregnant and immunocompromised hosts. Despite many efforts on its genetic characterization, an entirely clear picture of the population structure in Europe has not been achieved yet. The present study aimed to summarize the available genotyping information and to map the distribution of circulating strains. There is consensus on type II *T. gondii* genotypes prevailing in Europe, but the absence of harmonization in the use of typing methods limits detailed knowledge. Standardized, high-end typing tools and integrative strategies are needed to fill the gaps and complete an accurate image of the *T. gondii* genetic population in Europe.

## Introduction

**Figure 1:**
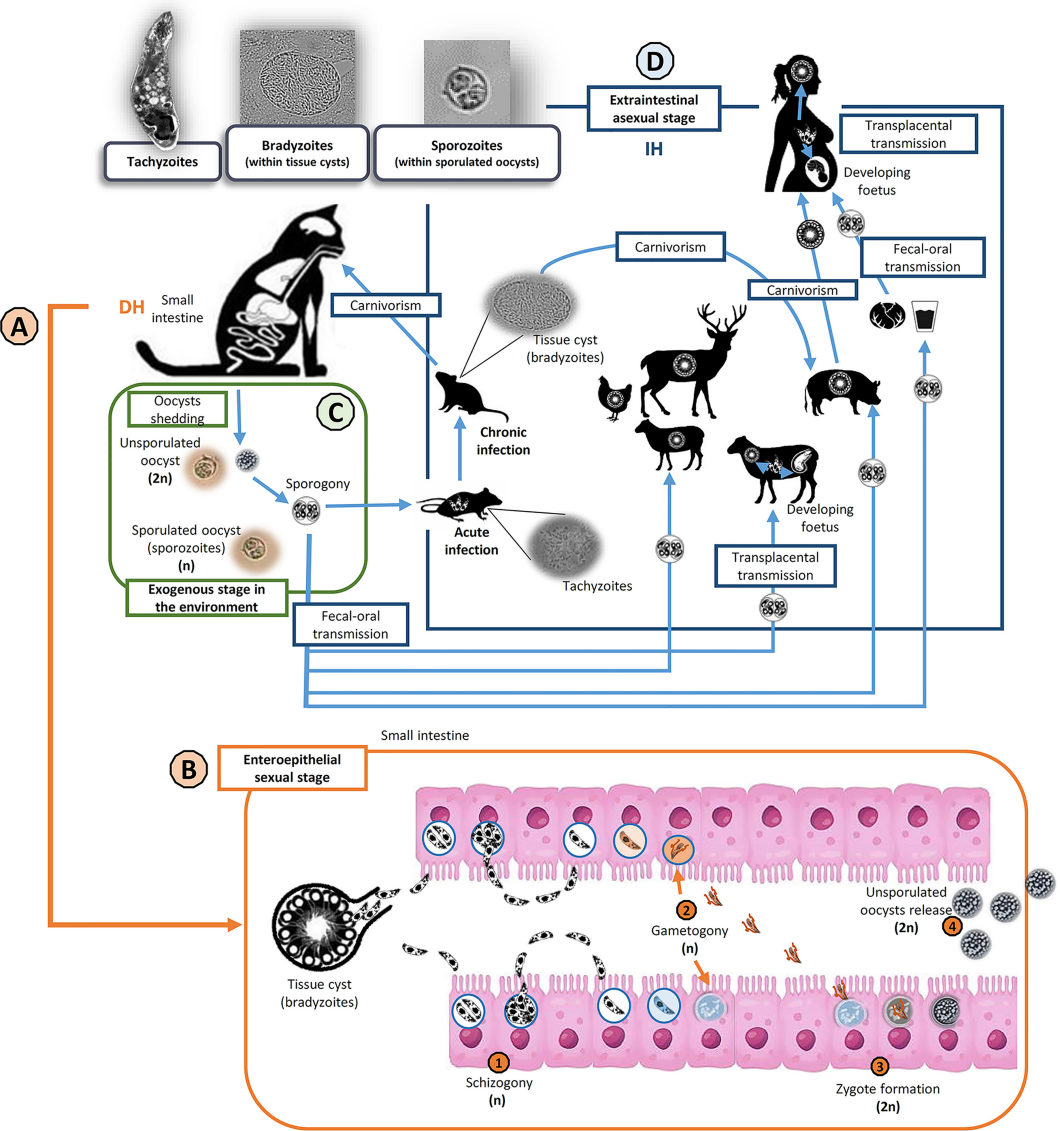
*Toxoplasma gondii* life cycle and transmission routes. Invasive stages in the life cycle of *T. gondii* (top left): tachyzoites, bradyzoites (inside tissue cysts) and sporozoites (within sporulated oocysts). Members of the family Felidae (domestic and wild cats) are the only known definitive hosts (DH) of *T. gondii* **(A)**. Cats become infected mainly after ingestion of viable tissue cyst. Then, the enteroepithelial sexual stage of the cycle takes part in the small intestine of DH **(B)**. After tissue cyst wall digestion and bradyzoites release (haploids, n), several asexual replication cycles by schizogony take place before gametogony begins. After fertilization of haploid macrogametes by haploid microgametes, resulting in diploid (2n) zygotes formation, an oocyst wall is developed around the parasite and epithelial cells lysis permits the release of the unsporulated oocysts to the lumen. During the exogenous stage of the cycle **(C)**, felids shed unsporulated non-infectious oocysts in their feces, but sporogony occurs in the environment within 5 days given suitable conditions of aeration, humidity, and temperature. Sporogony involves meiosis (postzygotic) and sporulation. Sporulated oocysts are infectious for both intermediate and definitive hosts. The extraintestinal asexual stage of the cycle **(D)** could occur in a wide variety of warm-blooded animals, as intermediate hosts (IH). IH can mainly get infected *via* fecal-oral transmission (ingestion of sporulated oocysts), carnivorism (ingestion of tissue cysts) or transplacental transmission (congenital infection). Usually, the infection courses asymptomatic and becomes chronic (tissue cyst formation), except in pregnant and immunocompromised host, when it could have serious clinical implications.

*Toxoplasma gondii*, the etiologic agent of toxoplasmosis, is an apicomplexan obligate intracellular protist of major medical and veterinary relevance. The complex life cycle of *T. gondii* is defined as facultative heteroxenous, with virtually all warm-blooded animals as intermediate hosts (including humans, domestic and wild mammals and birds), and members of the Felidae family acting as definitive hosts (Dubey, 2021a) (**Figure 1**). Toxoplasmosis is a zoonosis of global distribution (Robert-Gangneux and Dardé, 2012; Dubey, 2021a) and represents an excellent example of the One Health concept, since *T. gondii* is present and circulates through all compartments defined in this paradigm (Aguirre et al., 2019; Djurković-Djaković et al., 2019). Due to its wide host range the parasite is of importance not only in public health, but also in livestock industry and wildlife management programs. A FAO/WHO report considered *T. gondii* as the fourth most important foodborne parasite in the world (FAO and WHO, 2014). In addition, globalization and trade could contribute to the inter-regional and intercontinental spread of new parasite strains (Bertranpetit et al., 2017; Galal et al., 2019).

In humans, this parasite infects up to a third of the total global population (Bigna et al., 2020; Rostami et al., 2020). The infection is usually asymptomatic and results in chronicity; however, a primary infection in pregnant women could cause congenital transmission and consequent serious damage to the fetus (Jones et al., 2001). In immunocompromised individuals, severe neurologic and pulmonary clinical signs are frequently observed consequences of a re-activated or new infection (Wang et al., 2017; Robert-Gangneux et al., 2018). Finally, ocular toxoplasmosis is an increasingly recognized clinical issue in some parts of the world, also in immunocompetent patients (Shobab et al., 2013; Maenz et al., 2014).

In livestock, *T. gondii* infection is associated with significant economic losses linked to reproductive failure in several domestic species such as sheep and goats (Stelzer et al., 2019; Dubey et al., 2020a; Dubey et al., 2020b). Infection by *T. gondii* in livestock is also a risk to public health when animals destined for human consumption are involved (Opsteegh et al., 2016). Moreover, the parasite is a cause of concern in wildlife and zoo animals since *T. gondii* may cause lethal infection in particular species (Dubey, 2021a).

Although important oocyst-associated human toxoplasmosis outbreaks have been documented in the past few years (Pinto-Ferreira et al., 2019; Dardé et al., 2020; Dubey, 2021b), the relevance of the environmental route remains poorly investigated. *Toxoplasma gondii* oocysts have been detected in a wide spectrum of matrices worldwide, including fresh produce, water, soil or even bivalves (*e.g.*, mussels and oysters), which can accumulate *T. gondii* oocysts by water filtration (Shapiro et al., 2019; Marquis et al., 2019; Almeria and Dubey, 2021).

Strategies to reduce the disease burden of toxoplasmosis should be based on close collaboration between both medical practitioners and veterinarians under the One Health umbrella. The relative contributions of the different transmissible stages, sources, and transmission pathways (**Figure 1**) remain partly unknown. This lack of information on the attribution to specific infection sources has hampered the development of effective intervention strategies. That fact could be partly due to the absence of a systematic surveillance system for this zoonotic foodborne pathogen (van der Giessen et al., 2021). In addition, there are major geographical differences in the epidemiology of the infection as well as in food consumption habits around the world, which affect the importance of different transmission routes and specific food products for the occurrence of the infection (Galal et al., 2019).

In Europe, *T. gondii* is considered an important foodborne parasite that ranked high according to the multiple-criteria decision analyses (MCDA) (Bouwknegt et al., 2018) and disease-burden estimations for toxoplasmosis (Havelaar et al., 2015). Congenital toxoplasmosis is notifiable in 29 of 35 European countries surveyed, with routine testing of pregnant women in some countries such as Austria, Belgium, and France; nevertheless, underreporting is a major problem in most countries. ln animals, risk-based surveillance system of EU livestock needs to be improved to reduce human meat-borne infections; there is a lack of standardization and validation of diagnostic techniques as well as significant limitation in the number of animals tested and the information associated with them (*e.g.*, age and breeding system) (van der Giessen et al., 2021; EFSA and ECDPC, 2021).

Concerning the genetic diversity of *T. gondii* circulating in Europe, type II strains and, to a lesser extent, type III strains, are the dominating populations, both in domestic and wild environments (Khan et al., 2007; Lorenzi et al., 2016). However, the current globalization of trade seems to be causing risk situations that pose new research and public health challenges (Galal et al., 2019). For instance, cases of severe human toxoplasmosis have been reported in France due to the consumption of imported South and North American horsemeat contaminated with non-archetypal strains of the parasite (Elbez-Rubinstein et al., 2009; Pomares et al., 2011).

Because of the importance of a genetic characterization of *T. gondii* strains for epidemiological and clinical studies, this work is aimed to summarize present knowledge on the genetic population structure of *T. gondii* in Europe and the distribution of genotypes within the different compartments comprised in the One Health concept (*i.e.*, human, domestic and wild animals, and environment).

## *Toxoplasma Gondii*, A Complex Organism With Complex Genetics

### The *Toxoplasma gondii* Life Cycle, an Avenue for a Rich Genetic Diversity

A global distribution and a complex life cycle, including a sexual phase that makes genetic recombination events possible, have led to a wide genetic and phenotypic diversity within *T. gondii* populations circulating worldwide (**Figure 1**).

Almost all life cycle stages of *T. gondii* are haploid, with the exception of a short diploid phase from the zygote formation in the small intestine of felines (Martorelli et al., 2019) to sporulation in the environment, when haploid sporozoites are the result of a postzygotic meiosis (Dubey, 2021a) (**Figure 1**). Unlike for many apicomplexan parasites, the sexual phase is not mandatory in the case of *Toxoplasma* and *Neospora* genera and zoites can propagate by asexual replication indefinitely (Beck et al., 2009) (**Figure 1**).

During the 1990s, restriction fragment length polymorphism (RFLP) among other methods allowed researchers to establish the existence of three clonal lineages distinguished according to their virulence for mice. Type I isolates were 100% lethal to mice, irrespectively of the dose, while types II and III were moderately or non-virulent in a dose-dependent manner (Dardé et al., 1992; Sibley and Boothroyd, 1992; Howe and Sibley, 1995; Howe et al., 1996). Since then, global population structure and genetic variability of *T. gondii* has been extensively investigated. The rapid development of multilocus-sequencing methods, and the description of a wide panel of new PCR-RFLP and microsatellite (MS) markers led to solid observations on the predominance of three clonal/archetypal types or lineages in Europe and North America, but new concepts of “recombinant” and “atypical/non-canonical” strains appeared on the scene (Ajzenberg et al., 2002; Ajzenberg et al., 2005; Khan et al., 2005; Su et al., 2006; Khan et al., 2007). Later, pioneering long term activities in sampling *T. gondii* isolates world wide (*e.g.*, [Lehmann et al., 2006; Dubey et al., 2020c]), the establishment of a Toxoplasma Biological Resource Centre located in France (Rocaboy et al., 2020), or the release of the specific genomic database ToxoDB (http://ToxoDB.org), provided an excellent and continuing foundation for further population genetic analyses.

### An Eye on *Toxoplasma* Genome-Wide Aspects

The total haploid genome of *T. gondii* contains 13 chromosomes, with a total genome size of about 65 million base pairs (Mbp) and more than 8300 protein coding genes identified (Lorenzi et al., 2016; Xia et al., 2021). The genome-wide polymorphism rate between the three archetypal clonal lineages has been estimated to be approximately 1%, characterized by an extensive bi-allelism falling into type I, II and III single nucleotide polymorphisms (SNP) (Grigg et al., 2001; Khan et al., 2005; Boyle et al., 2006; Sibley and Ajioka, 2008). The origin of this clonality has been suggested to be due to a recent emergence from a common ancestor within the last 10,000 years during the domestication process of cats and various livestock species (Su et al., 2003). In addition, an extensive bypassing of the sexual cycle may have led to a continuous asexual propagation, resulting in rare possibilities for meiotic crosses between the highly similar parental strains (Sibley and Ajioka, 2008) only observed occasionally in naturally infected cats (Herrmann et al., 2012a). Nevertheless, this hypothesis is not applicable to the South American subcontinent, where a notably higher prevalence (and burden) of the infection, a larger spectrum of susceptible intermediate host species along with an increased diversity of wild felids might have promoted more frequent recombination events resulting in a contrasting, extremely diverse and largely non-archetypal population (Shwab et al., 2014; Bertranpetit et al., 2017).

### Global *Toxoplasma gondii* Population Genetic Structure

Until date there have been several comprehensive attempts to unravel the population structure of the parasite aided by great advances in molecular typing techniques. In an extensive and in-depth study based on phylogenetic analysis of above 950 typed isolates worldwide, 15 well-defined haplogroups were identified (Su et al., 2012), which were subsequently expanded to 16 and assorted into 6 major clades (clade A-F) based on whole genome sequencing analyses (Lorenzi et al., 2016).

The three clonal types dominating Europe and North America (corresponding to haplogroups (HG) 1, 2 and 3) were joined by a fourth clonal lineage (HG12) largely confined to North America, where it is more common in wild animals. In contrast, much greater genetic diversity is observed in South America, where the population seems to consist of a few major clonal complexes and abundant less related isolates (Khan et al., 2007; Pena et al., 2008; Khan et al., 2011; Jiang et al., 2018).

It has been suggested that African and Asian *T. gondii* populations could be a mixture between both above situations, with abundance of isolates belonging to type I, II, and III clonal lineages, coexisting with a considerable number of other recombinant or atypical genotypes, but exhibiting a less divergent character than in South America; however, both continents remain poorly explored, especially in tropical regions (Chaichan et al., 2017; Galal et al., 2018). With regard to the geographical origin of the species, paradoxically there are conflicting theories. On the one hand, a combination of molecular phylogenetic and phenotypic analyses suggested a North American common ancestor that entered South America and diversified there after reestablishment of the Panamanian land bridge (Khan et al., 2007; Minot et al., 2012). Nevertheless, subsequent phylogenetic and geostatistical approaches led to hypothesize a South American origin of *T. gondii* and its initial spread through North America, Asia, Europe and finally Africa, through different migration routes, linked to the co-evolution of Felidae family members and humans (Bertranpetit et al., 2017).

## *Toxoplasma Gondii* Genotyping Tools in Europe: Is there a Consensus?

**Figure 2:**
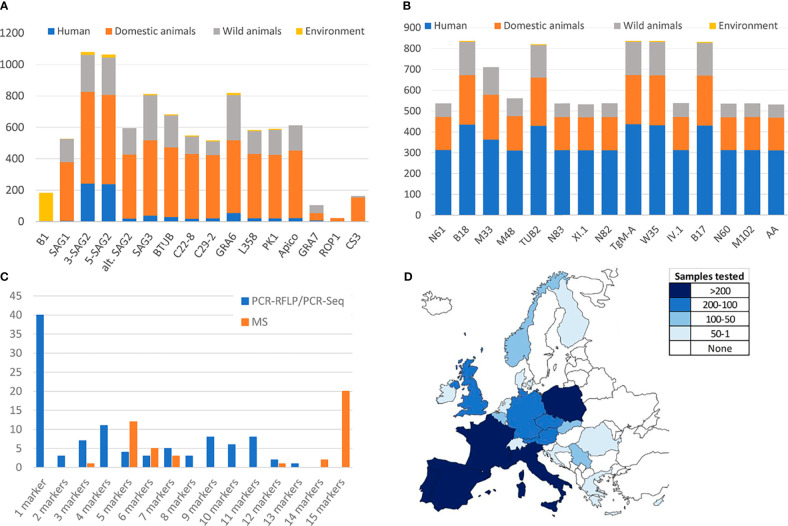
Analyses of the methodologies used within European *Toxoplasma gondii* genotyping studies. **(A)** Number of samples typed by each PCR-RFLP marker in genotyping European studies. **(B)** Number of samples typed by each MS marker in genotyping European studies. **(C)** Proportion of studies implementing different number of PCR-RFLP/PCR-Seq or MS genotyping markers. **(D)** Geographic distribution of genotyped samples across Europe. Only studies meeting the cut-off criterion (at least 4 PCR-RFLP/PCR-Seq or 5 MS markers applied) have been considered.

Available genotyping methodologies, PCR-RFLP, PCR-sequencing, MS-typing among others, have been irregularly applied in different areas, over different matrices and in a different manner by distinct research groups. The present section aims to examine the use of common methodologies within the European context. PubMed database was searched combining the terms “Toxoplasma gondii”, “genotyping”, “typing”, “type” and each different possible host designations or categories (*e.g.*, human, goat, fox, marine mammals, etc.) or environmental matrices (*e.g.*, water, soil, fresh produce, etc.) considered. Both *T. gondii* strain genotyping studies involving isolated viable parasites or DNA positive specimens/clinical samples from Europe were included. Nevertheless, data from overseas territories in other continents and zoo-kept animals were not covered, in order to better limit the origin of infections to continental Europe. Finally, 101 and 43 studies including PCR-RFLP/PCR-Sequencing or MS typing, respectively, were selected (see **Supplementary Tables S1, S2**). Despite the large number of studies aiming at a genetic characterization of European *T. gondii* strains, the data are limited due to several factors. After analysis of the extracted data, it seems to be apparent that there is a notable variance in the identity and number of markers used among the studies (**Figures 2A–C**). The selected studies comprised the use of up to 15 different PCR-RFLP (**Figure 2A**), PCR-Seq or MS (**Figure 2B**) markers. The use of an insufficient number of molecular markers may represent a problem because a large part of diversity might be missed or genotypically different parasites not efficiently distinguished. This is especially worrying in the case of PCR-RFLP and PCR-sequencing, since an important proportion (40%, 40/101) of these studies implemented a single-locus typing method, therefore involving major limitations for reliable strain classification (**Figure 2C**). The most frequently used marker was *SAG2* (5’ and 3’ ends of the gene) probably because it was among the first PCR-RFLP markers described, setting a milestone on *T. gondii* genetic studies (**Figure 2A**) (Sibley and Boothroyd, 1992; Howe and Sibley, 1995). On the other hand, comparison between studies is hardly possible if assays are based on infrequently used genes, such as *ROP1* (Haque et al., 1999; Turčeková et al., 2013), or on markers, like the *B1* gene, mostly applied in a certain type of environmental specimens (*i.e.*, water, soil, air, vegetables, or fruit) (**Figure 2A**) (Burg et al., 1989; Sroka et al., 2008; Sroka et al., 2009; Sroka et al., 2010). Regarding MS typing procedures, the number of markers has not been observed as a problematic issue since the use of five “genotyping” markers or the complete panel of eight “genotyping” plus seven “fingerprinting” MS markers is quite widely used (**Figure 2B**).

Furthermore, it is observed that regardless of the typing technique used, the collected information (from 21 different countries) is highly unbalanced between countries, and there is a lack of data for large areas of the European continent (**Figure 2D**). There are cases in which the same samples have been analyzed by different techniques (PCR-RFLP, MS and/or PCR-sequencing), with matching results although with of course different resolution power (Prestrud et al., 2008; Stajner et al., 2013; Verma et al., 2015).

**Figure 3:**
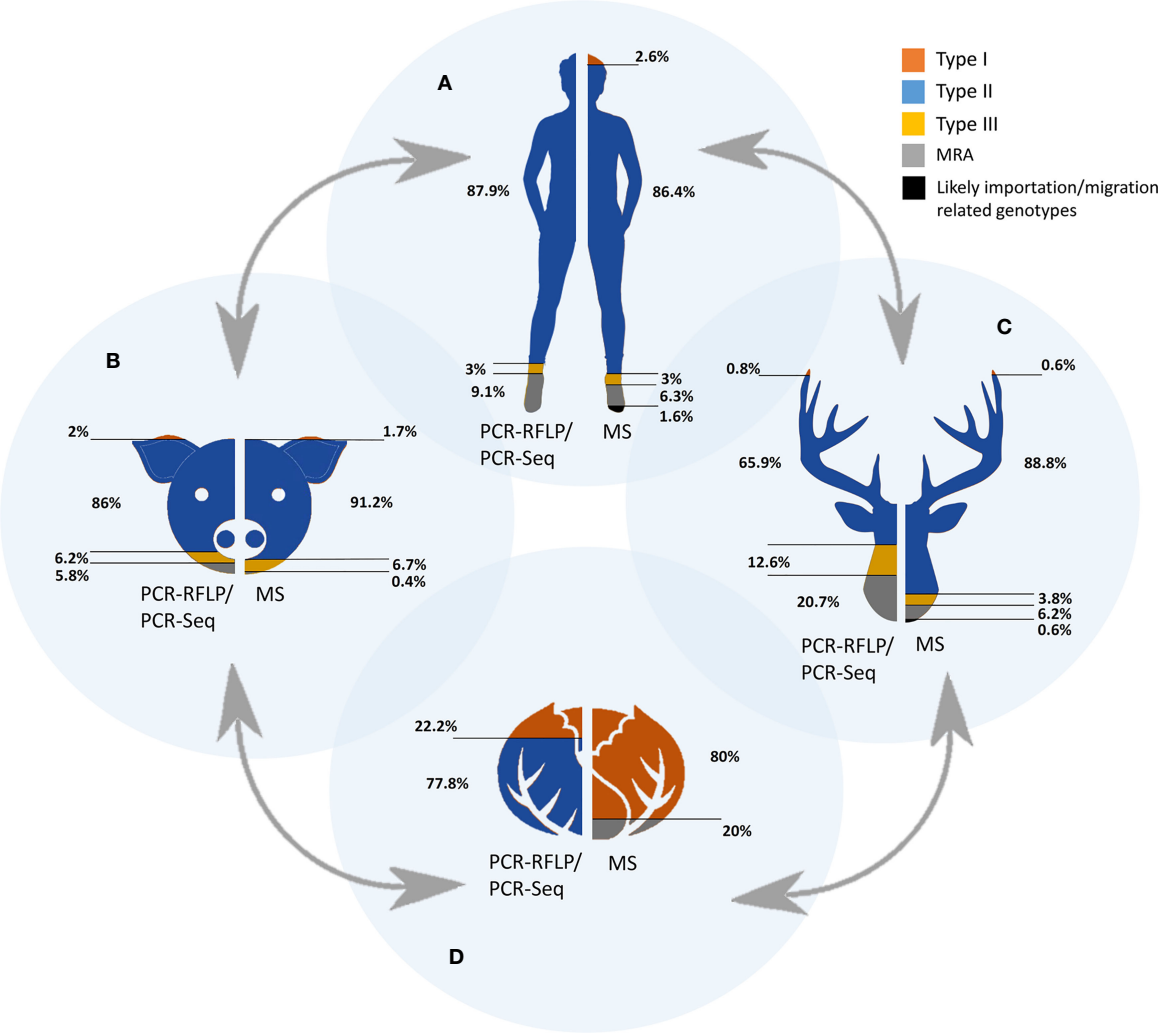
Occurrence of *Toxoplasma gondii* genetic types by each of the One Health compartments. The four interconnected compartments comprised in the One Health concept are represented as humans **(A)**, domestic animals **(B)**, wild animals **(C)** and environment **(D)**. Colored areas represent the proportion of each genetic types detected either by PCR-RFLP/PCR-Seq or MS-typing. MRA: mixed infections, recombinant and atypical genotypes detected.

Aiming to find the right balance between reliability and robustness, and taking into account the number of studies implementing a different number of PCR-RFLP/PCR-Seq or MS genotyping markers (**Figure 2C**), a minimum of four and five genomic regions analyzed was established as a “cut-off”, respectively. To this end, 51 (with typing results on n=804 samples) and 42 studies (n=831 samples typed) including PCR-RFLP/PCR-Seq or MS typing, respectively, were considered to represent a robust pan-European overview (**Table 1** and **Figure 3**).

**Table 1:** Prevalence of the *Toxoplasma gondii* genetic types observed in isolates and DNA positive specimens/clinical samples in Europe according to the four compartments within the One Health concept (human, domestic animals, wildlife, and environment) and based on PCR-RFLP/PCR-sequencing or MS data.

	Humans		Domestic animals^(1)^		Wildlife^(2)^		Environment^(3)^		TOTAL	
	RFLP/Seq (%)	MS (%)	RFLP/Seq (%)	MS (%)	RFLP/Seq (%)	MS (%)	RFLP/Seq (%)	MS (%)	RFLP/Seq (%)	MS (%)
**Type I**	0 (0)	11 (2.6)	10 (2)	4 (1.7)	2 (0.8)	1 (0.6)	2 (22.2)	4 (80)	14 (1.7)	20 (2.4)
**Type II^(4)^**	29 (87.9)	370 (86.4)	431 (86)	217 (91.2)	172 (65.9)	142 (88.8)	7 (77.8)	0 (0)	639 (79.5)	729 (87.7)
**Type III**	1 (3)	13 (3)	31 (6.2)	16 (6.7)	33 (12.6)	6 (3.8)	0 (0)	0 (0)	65 (8.1)	35 (4.2)
**MRA**	3 (9.1)	27 (6.3)	29 (5.8)	1 (0.4)	54 (20.7)	10 (6.2)	0 (0)	1 (20)	86 (10.7)	39 (4.7)
**Likely importation/migration related genotypes**	–	7 (1.6)	–	0 (0)	–	1 (0.6)	–	0 (0)	-	8 (1)
**TOTAL**	33	428	501	238	261	160	9	5	804	831

Percentages are given in brackets. MRA: Mixed infections and recombinant or atypical genotypes; Likely importation/migration related genotypes (Africa1, Caribbean2, Caribbean3); -: PCR-RFLP method is not valid for intra-genotype differentiation. Compartments: (1) livestock (poultry, cattle, small ruminants, equines, pigs) and pets (carnivores); (2) rodents, marine mammals, wild ungulates (Cervidae, Bovidae, swine), mesocarnivores, wild cats, and wild avian species; (3) water, soil, air, fresh produce, ticks, and bivalves; (4) PCR-RFLP profiles suggesting a type II PRU variant (type II alleles combined with type I allele at Apico marker) were included within Type II category.

## General Picture of The Genetic Population in Europe

PCR-RFLP and MS typing are the most widely used methods, but except for predominant lineages and some unique strains, equivalence between assigned genotypes by each technique remains at some extent confusing; thus, remarks will be given separately. The classification of an isolate into archetypal, recombinant or atypical, or even distinguishing between a recombinant strain and a mixed infection (co-infection) is a sensitive issue. In most cases this requires the availability of viable parasites in a sample that could be separated into different co-existing clonal populations, *e.g*., by limiting dilution cloning (Herrmann et al., 2010). The unambiguous identification of mixed infections is difficult in only DNA positive materials and largely depends on the number and the discriminating power of markers used for genotyping. Therefore, from a critical viewpoint, mixed infections, as well as infections with recombinant (mixture of type I, II or III alleles as a consequence of recombination events) and atypical (including unique polymorphisms at any loci) strains should be treated as a whole (MRA category), differentiating them from the widely prevalent archetypal clonal strains (*e.g.*, types I, II and III). Based on the One Health concept, we sorted genotypic information according to samples or isolates origin into four “compartments”, namely humans, domestic animals, wildlife, and environment (**Table 1** and **Figure 3**).

### *Toxoplasma gondii* Genetic Diversity Based on PCR-RFLP or PCR-Sequencing Methodologies

Concerning strain types detected in humans, only three countries are represented (Germany, Poland and Serbia) in five studies with a total of 33 samples typed (Djurković-Djaković et al., 2006; Nowakowska et al., 2006; Stajner et al., 2013; Marković et al., 2014; Herrmann et al., 2014). Among them, almost 90% (29/33) corresponded with type II strains, only one type III was detected, and MRA infections were described in three cases. The presumed predominance of type II in Europe is evident but non-conclusive since data could be representative only of central Europe.

Most European (geno)typed samples have been collected from infected domestic (pets and livestock) and wild animals. Regarding domestic animals, the range of countries represented is wider but not enough, with molecular studies from Austria, Czech Republic, Denmark, France, Germany, Ireland, Italy, Poland, Portugal, Serbia, Spain, Switzerland, and The Netherlands (22 studies with a total of 501 samples) (**Table 1** and **Supplementary Table S1**). Likewise, studies could be sorted according to the host, including data from sheep, goat, cattle, pig, horse, chicken, dog, and cat, standing out chicken and pig species in terms of sampling effort, with 102 and 76 samples typed, respectively. Type II strains were reported in 86% (431/501) of samples, together with 6.2% (31/501) of type III, 2% of type I (10/501) and approximately 6% (29/501) of MRA infections (**Table 1** and **Figure 3**). Concerning wild animals, European studies include data from Croatia, Czech Republic, Denmark, Germany, Italy, Norway, Poland, Serbia, Spain, and the UK, with a total of 261 samples collected in 25 different studies. It involves data from a wide variety of hosts such as rodents, marine mammals, wild cats, wild swine, mesocarnivores, wild ruminant ungulates, and wild avian species. Within the group of wild animals, mesocarnivores were those with the highest number of studies (n=8) and samples analyzed (n=144). Approximately 66% of strains circulating in wildlife were reported to be type II (172/261), 20.7% MRA (54/261), 12.6% type III (33/261), and 0.8% type I (2/261) (**Table 1** and **Figure 3**).

Regarding genotypes present in environmental samples, the situation is even more restricted, with only two studies having met the requirements accounting for a total of nine samples. Type II strains were reported in seven samples of vegetables in the Czech Republic (Slany et al., 2019) whereas type I alleles were observed in DNA extracted from two ticks (*Dermacentor reticulatus*) collected in field areas of Poland (**Table 1**) (Wojcik-Fatla et al., 2015).

As a whole, literature data on PCR-RFLP typing or PCR-sequencing suggest a clear predominance of type II strains circulating in Europe, that comprises of 79.5% (639/804) of the total samples collected in 51 different studies included (**Table 1**). Previous serotyping studies largely corroborated this type II predominance (Nowakowska et al., 2006; Morisset et al., 2008; Maksimov et al., 2012). Reports on type I strains are truly scarce, 1.7% (14/804) of samples, whereas type III strains seem to be responsible for 8.1% of total samples (65/804). Finally, MRA infections were reported for 10.7% (86/804) of the records. Despite the limitation on the data, it could be pointed out the higher burden of type III strains and MRA infections in the case of wildlife animal species in comparison with the rest of European compartments considered. In **Figure 2D**, geographic distribution of genotyped samples across Europe is represented. Germany, Italy, and Serbia are the countries with the highest number of PCR-RFLP/PCR-Seq based genotyping investigations.

**Figure 4:**
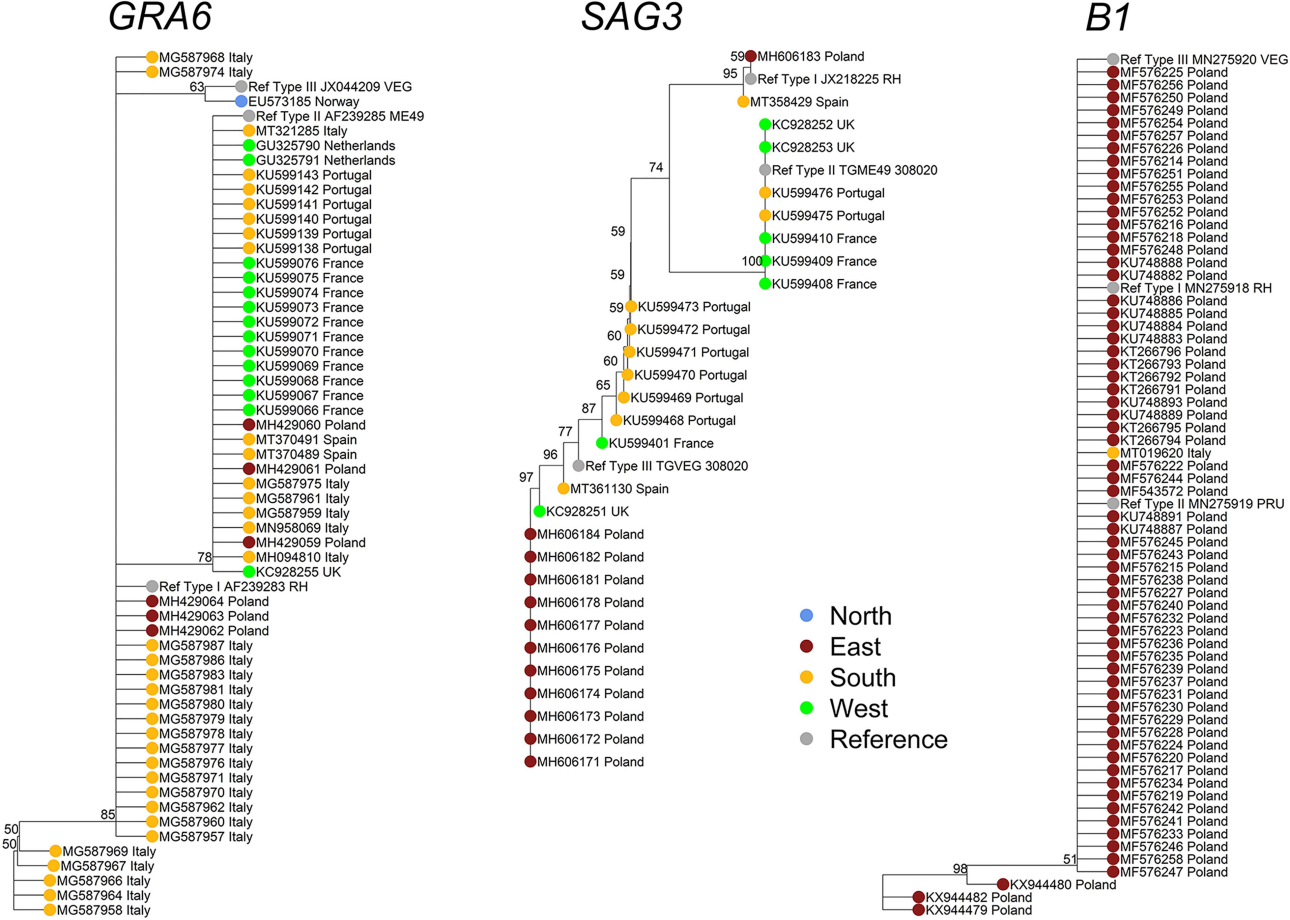
Phylogenetic analyses of the *Toxoplasma gondii* population in Europe based on available *GRA6*, *SAG3*, and *B1*-derived sequences. To analyze the genetic population of *T. gondii* in Europe based on the nucleotide sequences of different *T. gondii*-specific markers, the respective entries were downloaded from the NCBI nucleotide database (https://www.ncbi.nlm.nih.gov/nucleotide?cmd=search) using the R package “rentrez”. In the first step, using a search string (*e.g.*, “Toxoplasma[ORGN] AND GRA6[ALL]”), all available data on the respective markers were downloaded by the R function “rentrez::entrez_search” and “rentrez::entrez_summary”. A total of 7776 entries were identified and downloaded worldwide. From these data, but after a detailed literature screening and manual curation of the dataset, only nucleotide entries from European isolates (n=501) were extracted and annotated accordingly (**Table S3**). However, for quality reasons as well as due to low number of available sequences per respective marker, several sequences and markers were excluded from the analysis (**Table 2**). To this end, 76 nucleotide sequences from *B1* gene, 86 from *GRA6* and 49 from *SAG3* loci were downloaded as an independent multifasta data file for each selected *T. gondii* typing marker. The download was performed using the R function “rentrez::entrez_fetch”. Alignment of the respective nucleotide sequences with calculation of the Tamura-Nei genetic distance and generation of the Neighbor-Joining trees was performed using Geneious Prime^®^ 2021.1.1 [build 2021-03-12 13:25 Java version 11.0.9 + 11 (64 bit)]. The trees were exported in “newick” format and then modified with the R packages “base”, “ape”, “dendextend” and “castor”. To simplify the view of the trees, the resolution was reduced using the r function “castor::collapse_tree_at_resolution” by applying the cut-off in the “resolution” parameter for each tree. This parameter specifies the phylogenetic resolution at which the tree should be collapsed. This is the maximum distance a descending tip can be from a node such that the node collapses into a new tip. If set to 0 (default), only nodes whose descending tips are identical to the node will be collapsed; finally, 56, 29, and 65 sequences were analyzed for *GRA6*, *SAG3*, and *B1*, respectively. Thus, resolution cut-offs of 0.008 (GRA6 tree), 0.005 (SAG3 tree) and 0.004 (B1 tree) were applied respectively.

Complementarily, we proposed the use of sequencing data from *Toxoplasma* molecular markers deposited in NCBI database (https://www.ncbi.nlm.nih.gov/nucleotide?cmd=search) to implement possible phylogenetic analyses. After a detailed screening and manual curation of nucleotide sequences available from *T. gondii* specific genetic markers (n=7776), only entries from Europe (n=464; 6%) were extracted (**Table 2** and **Supplementary Table S3**). Then, the only markers that had sufficient high-quality sequences from at least four different European countries to perform a robust phylogenetic analysis were loci *GRA6* (n=86) and *SAG3* (n=49). In addition, *B1* gene (n=76) was included as it is the marker that better represents the environmental compartment (**Supplementary Table S3**). Sequences were downloaded and assessed further (**Figure 4**). The composition of dendrograms obtained was not related to the geographical origin of sequenced *T. gondii* strains, and sequences were barely allocated to defined clusters along with respective type I, II and III canonical references. *GRA6* and *SAG3* sequences seem to provide a higher resolution than *B1* sequences, without discriminative power to split major lineages into separated clusters.

**Table 2:** Summary on sequence data available in GenBank for European *Toxoplasma gondii* isolates and samples.

Marker or gene	Country								Total
	France	Italy	The Netherlands	Norway	Poland	Portugal	Spain	United Kingdom	
***Apico***		1							1
***B1***	1	6			69				76
***BTUB***		3			4			1	8
***c22-8***		5							5
***c29-2***		1							1
***CS3***							2		2
***GRA6***	12	35	2	1	19	12	3	2	86
***GRA7***	33					12	3		48
***PK1***		1							1
***ROP8***	1								1
***SAG1***	12	3			17				32
***SAG2***						118			118
***3’-SAG2***		1			3			1	5
***5’-SAG2***		4						1	5
***alt. SAG2***		22			3				25
***SAG3***	12	1			14	12	5	5	49
***UPRT-intron1***				1					1
**Total**	71	83	2	2	129	154	13	10	464

Available sequences were further analyzed when four or more countries were covered. B1 gene was also included (data from three countries) as it is the marker that better represents the environmental compartment. Sequences likely related to migration/importation, reference strains, or with sequencing errors were excluded.

### *Toxoplasma gondii* Genetic Diversity Based on MS Methodologies

Under the view of the available literature (**Supplementary Table S2**), the number of samples typed by less than 5 MS loci is negligible compared to the 831 samples typed in 42 different studies by using five or more MS markers (**Table 1**). Apart from type I, II, III or MRA infections, by MS typing it was also possible to identify specific genotypes such as *Africa1*, *Caribbean2*, *Caribbean3* even characterizing only five loci (*B18*, *TUB*, *Tg-MA*, *W35* and *B17*).

Unlike the previously mentioned methods, the MS-based methodology has been widely used in the genetic characterization of human samples, involving a total of 428 samples in 20 different studies. Despite the participation of a greater number of European countries, France clustered 77.3% of the human samples analyzed (Ajzenberg et al., 2009; Ajzenberg et al., 2015), followed by Portugal (11.7%) (Ajzenberg et al., 2009; Vilares et al., 2017), Denmark (4.7%) (Jokelainen et al., 2018), and Belgium (4.4%) (Gisbert Algaba et al., 2020); most of the other countries contributed with up to three single isolates (Austria, Germany, Romania, Serbia, The Netherlands, and UK). Concerning strain types detected in human population, 86.4% corresponded with type II strains, the types I and III were found in low proportions (2.6 and 3% respectively), and those of MRA infections corresponded to 6.3% of cases. In addition, six cases of human infection with *Africa1* strains and one case with *Caribbean2* were detected in France, Denmark, and Belgium (Ajzenberg et al., 2010; Fekkar et al., 2011; Su et al., 2012; Jokelainen et al., 2018) (**Table 1** and **Figure 3**). The predominance of type II in Europe is again clear but once more it should be borne in mind that extensive areas of the continent are still not represented.

The second most studied compartment was that of domestic animals, involving a total of 238 samples in 15 different investigations. Once again, France (36.9%) and Portugal (20.6%), together with Austria (27.3%), stood out in the number of genotyped samples. Data from Finland, Germany, Italy, Romania, Serbia, and The Netherlands are also available. In respect of the different hosts studied, most of the samples were collected from chicken (93) and sheep (91) (Verma et al., 2015; Bertranpetit et al., 2017; Shwab et al., 2018). In pets and livestock, type II strains were reported in 91.2% (217/238) of samples, along with a 6.7% (16/238) of type III and 1.7% of type I (4/238). Apart from that, only one sample presented a MRA profile (0.4%, 1/238). Concerning wildlife, European studies included data from Belgium, Czech Republic, England, Finland, France, Italy, Norway, Portugal, Serbia, and Spain, with a total of 160 samples collected in 15 different publications; a wide variety of hosts were included in such surveys, highlighting red foxes (*Vulpes vulpes*) (n=54) (Aubert et al., 2010; De Craeye et al., 2011) and wild boars (*Sus scrofa ferus*) (n=44) (Richomme et al., 2009; Gisbert Algaba et al., 2020). Among strains circulating in wild animals, 88.8% corresponded to type II (142/160), 6.2% (10/160) to MRA infections and 3.8% (6/160) to type III. Only one case of type I and another of a *Caribbean3* genotype were reported (0.6% each, 1/160) from a pigeon from Portugal and a wild boar from Italy, respectively (Vilares et al., 2014; Sgroi et al., 2020).

As occurred in previous section regarding PCR-RFLP and PCR-sequencing based studies, typing reports on environmental samples again are quite rare. One study reported genotyping results from Mediterranean mussels (*Mytilus galloprovincialis*) collected in southern Italy (Santoro et al., 2020), with four samples surprisingly belonging to type I and one sample typed as a recombinant or mixed profile. As this is the only study, including such a small sample size, general conclusions cannot be reached at this time.

On balance, the prevalence figures obtained from reviewing the available data on *T. gondii* strains genotyped by MS in Europe are quite similar to those obtained by PCR-RFLP and PCR-sequencing methods. The predominance of type II strains in Europe is again evident, involving 87.7% (729/831) of the total samples analyzed in 42 studies that meet the criteria of at least 5 genotyping markers characterized (**Table 1** and **Figure 3**). Type I strains remain infrequent, representing 2.4% (20/831) of samples. On the other hand, the prevalence of type III and non-assorted, recombinant strains or mixed infections were slightly lower compared to PCR-RFLP and PCR-sequencing methods with almost 4.2% (35/831) and 4.7% (39/831) of total records, respectively. Finally, MS-typing was able to resolve other non-canonical haplogroups, *i.e.*, *Caribbean1*, *Caribbean3* or *Africa1*, allowing to identify *T. gondii* strains possibly imported to Europe (1%, 8/831), either by human migration or trade. Overall, France, Portugal, Austria, and Belgium are the countries with the highest number of MS genotyping results; in contrast, there are large areas of the continent from which there is no information, especially northern European countries (**Figure 2D**).

**Figure 5:**
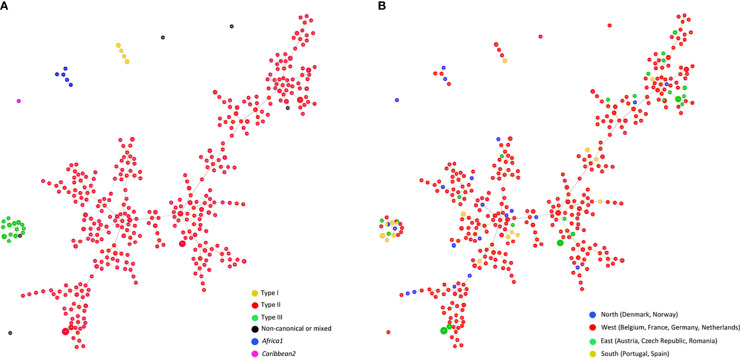
Global optimal eBURST analysis using the Full-MST (goeBURST distance) option of European *Toxoplasma gondii* samples typed by 15 MS regions. In total, n = 487 isolates microsatellite typed at all 15 microsatellite markers were included, representing n=384 separate microsatellite types. Applying a Locus Variant Level 4, types clearly separated into four major groups; each circle represents an individual type; size of circles correlates with total number of samples with identical profile. **(A)** Distribution by genotypes of the population, distinguishing between Type I, II, III, non-canonical or mixed patterns, *Africa1*, and *Caribbean2* types. **(B)** Geographical distribution of the population distinguishing between samples from North, West, East or South Europe. Type I, type III and non-canonical *T. gondii* types, including also a group representing *Africa1* type, are clearly separated from Type II while no clear regional patterns can be observed in Type II *T. gondii* samples.

A Global optimal (go)eBURST Full MST (goeBURST distance) analysis (Feil et al., 2004; Francisco et al., 2009) of all *T. gondii* DNA samples typed by 15 MS markers (n=487) using PHYLOViZ 2.0a (http://www.phyloviz.net/) was performed. At Locus Variant Level 4 the minimum spanning tree-like structure clearly separated type I, type II, type III and MRA genotyping results (**Figure 5A**). Within the type II group a high level of diversity was observed. There seems to be no clear regional pattern, separating type II samples from different parts of Europe (*e.g.*, the northern part, Denmark, Norway and the eastern part, Austria, Czech Republic, Romania), as shown in **Figure 5B**. This is only partially in accord with results reported in France for *T. gondii* strains involved in human toxoplasmosis where in rural regions *T. gondii* associated with cases of congenital toxoplasmosis were genetically different between the eastern and western part of the country based on MS typing results (Ajzenberg et al., 2015).

## Integrative Analysis: Evidence from a Pan-European Perspective

The complex biology and epidemiology of *T. gondii* means that researchers face not only the detection of routes or sources of transmission, as in other emerging zoonotic diseases, but also the enormous variety of susceptible hosts that makes it an underestimated and silent concern, only visible in specifically vulnerable groups of populations (immunosuppressed or pregnant hosts). This review examines the distribution of various *T. gondii* genotypes throughout the European continent taking into account the different One Health compartments. As a whole, the predominance of clonal type II strains is evident, but exhaustive published data collection and analysis suggests the existence of an interesting proportion of divergent strains (MRA), slightly more concentrated in the wildlife compartment. Hence, the dichotomy “domestic *versus* wild” so manifest in the American continent is possibly present in Europe as well (Mercier et al., 2011; Jiang et al., 2018; Galal et al., 2019). Nonetheless, the potential genetic diversity of *T. gondii* in wildlife has been less studied than in domestic animals, with fewer samples available, with less effort/success on parasite isolation and consequently limited PCR amplification and a limited resolution of typing assays (Herrmann et al., 2012b; Verin et al., 2013; Bacci et al., 2015; Uzelac et al., 2019). If only studies in which the isolation of the parasite was achieved (mainly in mice or cell culture) and where a sufficient number of RFLP or MS genotyping markers were applied are taken into account, the genotypes described are mainly clonal type II. On the other hand, the selection of certain strains at the expense of others during isolation procedures has been demonstrated in literature (Verma et al., 2017; Fernández-Escobar et al., 2020a). Therefore, data obtained directly from clinical samples should not be ignored but need verification, and conclusions should be drawn with caution. In short, findings should be always interpreted cautiously, as well as with interest, since strains that circulate in wildlife are a source of infection for domestic animals and humans, and have been associated with greater pathogenicity at least in North and South America (Dubey et al., 2014). Virulence characterization data of European field *T. gondii* strains are worryingly scarce (Uzelac et al., 2020; Fernández-Escobar et al., 2021).

Clonal type III-related strains were also highlighted, mainly detected in animal hosts. Some authors claimed that type III alleles are more frequently detected in southern Europe compared to other parts of the continent (Kuruca et al., 2019; Uzelac et al., 2021), but the reality is that France, Italy, and Portugal are the countries that have published the most *T. gondii* genotyping studies, with a lower contribution from northern countries (**Figure 2D**), implying large areas without information. Type I alleles are particularly underrepresented in Europe. Most articles describing type I alleles during genotyping (Turčeková et al., 2013; Papini et al., 2015; Mancianti et al., 2015; Battisti et al., 2018; Santoro et al., 2020; Sroka et al., 2020) only involved direct genotyping from tissue samples DNA, with an often lower success in the amplification of typing markers. On the other hand, two studies (Verma et al., 2015; Moskwa et al., 2017) showed a complete clonal type I profile in two isolates obtained from an aborted bovine fetus in Portugal [firstly reported by (Canada et al., 2002)] and from an aborted fetus of European bison (*Bison bonasus bonasus L.*) in Poland, respectively. Clonal type I isolates fully typed by 15 MS markers have been also described infecting humans (Ajzenberg et al., 2010).

Standardization of typing methods is definitively necessary for the integration of genetic data. The BRC biobank (Biological Resource Center for Toxoplasma, www.toxocrb.com) was one of the approaches that comes closest to this objective, storing around 1500 strains from different hosts (humans or animals) and from different countries around the world, all genotyped by the widely applied 15 MS markers (Ajzenberg et al., 2010; Rocaboy et al., 2020). There are important limitations of traditional methodologies used for *T. gondii* typing, because only quite specific and restricted sites within a large *T. gondii* genome are assessed. Whole-genome sequencing (WGS) data analysis has emerged as the most suitable approach for a thorough analysis of the genetic diversity in *T. gondii*, its evolutionary history, and population structure. Although WGS is difficult to apply as a routine technique for strain typing, the number of studies using this technology is growing rapidly, mainly due to its enormous potential and the continued costs reduction. WGS data are publicly available only for a few isolates from Europe (namely PRU, MAS, FOU, BOF, TgH26044, TgH21016, TgH20005, Cz-H3, among others) of which only two (PRU, Cz-H3) belong to the dominant clonal type II. The others, although isolated in Europe (*i.e.*, France, Belgium) are at least partially reminiscent of strains likely originating from other continents, like FOU and BOF (Africa) or MAS (South America) (Lorenzi et al., 2016). The European Type II isolate PRU (Pruginaud) was assorted to Clade D, a clade which was established based on WGS data and comprises, in addition to other type II strains, of North American HG12 strains and some atypical North or South American strains (Lorenzi et al., 2016). The generation of WGS data on further strains including European type II strains could help to better understand the real genetic diversity within the dominant European strains, to explore the possible exchange of sequence blocks between clonal lineages in Europe and probably to link genetic differences not covered by the traditional widely used typing methods with phenotypic differences (*e.g.*, virulence in mice) evidenced in literature between European isolates (Fernández-Escobar et al., 2020b; Fernández-Escobar et al., 2021).

## Outstanding Questions

Under the light of the data exposed, authors identified some key questions that should be addressed:

- To what extent are the different anthropogenic factors involved in shaping *T. gondii* population structure in Europe?
- Is there an unexplored *T. gondii* biodiversity in the wild in Europe?
- Are traditional typing methods (PCR-RFLP, MS-typing) going to be replaced by Next-Generation or Third-Generation Sequencing techniques?
- Will Whole Genome Sequencing of European *T. gondii* help to understand differences in virulence?

## Conclusions

Despite many important efforts on *T. gondii* genotyping in Europe, the situation is still blurred and in need of extra and closer look. Still many questions remain unsolved and will constitute medium term challenges for researchers. Some important facts, like the lack of consensus over the methodologies and markers applied, the huge differences in samples’ quality and concentration, the sampling disparities among regions and the fact that vast areas remain unexplored, as well as the scarcity of data from human cases and environment, are the main limitations to having a complete picture. In this respect, epidemiological surveillance systems must be strengthened at many levels, in humans and in livestock industry (for example on farms, slaughterhouses, and during veterinary inspection of hunted and home slaughtered animals). Therefore, close collaboration between the medical and veterinary sectors is crucial.

There is consensus on type II *T. gondii* prevailing in Europe, followed by type III, but the presence of a noticeable proportion of recombinant and atypical genotypes whose phylogenetic positioning remains obscure, deserves further investigation. Standardized, high-end typing tools and integrative strategies within the One Health approach are needed to fill the existing gaps and provide a clear picture of the *T. gondii* population in Europe.

## Author Contributions

All authors listed have made a substantial, direct, and intellectual contribution to the work, and approved it for publication.

## Funding

MF-E was funded by UCM-Santander/2017 pre-doctoral Grants. MF-E, GS, PM, MJ, LO-M, and RC-B are part of the TOXOSOURCES consortium supported by the funding from the European Union’s Horizon 2020 Research and Innovation Programme under the Grant Agreement No 773830: One Health European Joint Programme.

## Conflict of Interest

The authors declare that the research was conducted in the absence of any commercial or financial relationships that could be construed as a potential conflict of interest.

The handling Editor OD declared a past collaboration with the author GS.

## Publisher’s Note

All claims expressed in this article are solely those of the authors and do not necessarily represent those of their affiliated organizations, or those of the publisher, the editors and the reviewers. Any product that may be evaluated in this article, or claim that may be made by its manufacturer, is not guaranteed or endorsed by the publisher.
