# Supplementary material for: Toxoplasma gondii Genotyping: A Closer Look Into Europe
Source: Front Cell Infect Microbiol. 2022 Mar 23;12:842595. doi: 10.3389/fcimb.2022.842595 (PMC8984497; doi:10.3389/fcimb.2022.842595)
Supplement: Supplementary file 1 [file Table_1.docx]

| **Table S1.** Data extracted from available studies on European *Toxoplasma gondii* strains PCR-RFLP and PCR-sequencing genotyping deposited in PubMed database (n=101). Typing results on both, isolated viable parasites and DNA positive specimens/clinical samples have been considered; data from overseas territories and zoo-kept animals were not included. | | | | | | | | | | | | | | |
| --- | --- | --- | --- | --- | --- | --- | --- | --- | --- | --- | --- | --- | --- | --- |
| **Compartment** | **Order** | **Host** | **Species** | **Country** | **Molecular markers** | **Markers**  **Max (Min)** | **Isolation** | **Type I** | **Type II** | **Type III** | **MRA** | **ND** | **TOTAL** | **Reference** |
| Human | Primates | Human | *Homo sapiens* | Denmark | PCR-Seq (GRA6) | 1 (1) | NO | - | 2 | - | - | - | 2 | [S1] |
|  |  |  |  | France | HRM (B1) | 1 (1) | NO | - | 34 | 29 | - | - | 63 | [S2] |
|  |  |  |  | France | Pyrosequencing (GRA6) | 1 (1) | NO | - | 1 | - | - | - | 1 | [S3] |
|  |  |  |  | France | PCR-RFLP (SAG2 (5'-SAG2 and 3'-SAG2)) | 1 (1) | NO | 7 | 55 | 6 | - | - | 68 | [S4] |
|  |  |  |  | France | PCR-RFLP (SAG1, ROP1 (5'-ROP1 and 3'-ROP1)) | 1 (1) | YES | - | 1 | - | - | - | 1 | [S5] |
|  |  |  |  | France | PCR-RFLP (SAG2 (5'-SAG2 and 3'-SAG2)) | 1 (1) | YES | 14 | 69 | 7 | - | - | 90 | [S6] |
|  |  |  |  | Germany | PCR-RFLP (altSAG2, SAG3, BTUB, GRA6, c22-8, c29-2, L358, PK1 and Apico) | 9 (1) | NO | - | 25 | 2 | 1 | - | 28 | [S7] |
|  |  |  |  | Greece | PCR-RFLP (GRA6) | 1 (1) | NO | - | 4 | 14 | - | - | 18 | [S8] |
|  |  |  |  | Greece | PCR-RFLP (SAG2 (5'-SAG2 and 3'-SAG2)) | 1 (1) | NO | 1 | - | 3 | 2 | - | 6 | [S9] |
|  |  |  |  | Italy | PCR-RFLP (altSAG2 and PK1) | 2 (2) | NO | - | 1 | - | - | - | 1 | [S10] |
|  |  |  |  | Poland | PCR-RFLP (SAG2 (5'-SAG2 and 3'-SAG2), SAG3, BTUB and GRA6) | 4 (4) | NO | - | 9 | - | - | - | 9 | [S11] |
|  |  |  |  | Poland | PCR-mini seq NTS2 | 1 (1) | NO | 59 | - | - | - | - | 59 | [S12] |
|  |  |  |  | Portugal | PCR-Seq (SAG2 (5'-SAG2 and 3'-SAG2) | 1 (1) | YES | 6 | 32 | - | 10 | - | 48 | [S13] |
|  |  |  |  | Serbia | PCR-RFLP (SAG2 (5'-SAG2 and 3'-SAG2), SAG3, BTUB and GRA6) | 4 (4) | YES | - | 1 | - | - | - | 1 | [S14] |
|  |  |  |  | Serbia | PCR-RFLP (SAG1, SAG2 (5'-SAG2 and 3'-SAG2), GRA6 and GRA7 (5`-GRA7 and 3`-GRA7)) | 4 (1) | YES | 1 | 2 | - | 1 | - | 4 | [S15] |
|  |  |  |  | Serbia | PCR-RFLP (SAG1, SAG2, GRA6 and GRA7) | 4 (4) | NO | - | - | - | 1 | - | 1 | [S16] |
|  |  |  |  | Slovakia | PCR-RFLP (SAG2 (5'-SAG2 and 3'-SAG2)) | 1 (1) | NO | 1 | - | - | - | - | 1 | [S17] |
|  |  |  |  | Spain | PCR-RFLP (SAG2 (5'-SAG2 and 3'-SAG2)) | 1 (1) | NO | 10 | 10 | 5 | - | 8 | 33 | [S18] |
|  |  |  |  | UK | PCR-RFLP (SAG3 and GRA6) | 1 (1) | NO | 2 | - | 2 | - | - | 4 | [S19] |
|  |  |  |  | UK | PCR-RFLP and PCR-Seq (SAG2 (5'-SAG2 and 3'-SAG2)) | 1 (1) | NO | 10 | 11 | 1 | 10 | - | 32 | [S20] |
| **Compartment** | **Order** | **Host** | **Species** | **Country** | **Molecular markers** | **Markers**  **Max (Min)** | **Isolation** | **Type I** | **Type II** | **Type III** | **MRA** | **ND** | **TOTAL** | **Reference** |
| Domestic animals | Perissodactyla | Donkey | *Equus asinus* | Italy | PCR-RFLP (SAG2 (5'-SAG2 and 3'-SAG2), altSAG2, SAG3, BTUB, c22-8, c29-2, GRA6, L358, PK1 and Apico) | 8 (4) | NO | - | 1 | 5 | - | - | 6 | [S21] |
|  |  | Horse | *Equus ferus caballus* | Italy | PCR-RFLP (SAG1, SAG2 (5'-SAG2 and 3'-SAG2), SAG3, c22-8, c29-2, GRA6, L358 and PK1) | 7 (5) | NO | 1 | - | 1 | 1 | - | 3 | [S22] |
|  | Artiodactyla | Goat | *Capra hircus* | Italy | PCR-RLFP (SAG1, SAG2 (5'-SAG2 and 3'-SAG2), altSAG2, SAG3, BTUB, c22-8, c29-2, GRA6, L358, PK1 and Apico) | 10 (6) | NO | 1 | - | 4 | 5 | - | 10 | [S23] |
|  |  |  |  | Italy | PCR-Seq (B1) | 1 (1) | NO | - | 3 | - | - | - | 3 | [S24] |
|  |  |  |  | Italy | PCR-RFLP and PCR-Seq (GRA6) | 1 (1) | NO | - | 1 | - | - | - | 1 | [S25] |
|  |  |  |  | Poland | PCR-RFLP and PCR-Seq (SAG1, SAG2 (5'-SAG2 and 3'-SAG2), SAG3, BTUB and GRA6) | 3 (1) | NO | - | - | 21 | - | 4 | 25 | [S26] |
|  |  |  |  | Portugal | PCR-Seq (SAG2 (5'-SAG2 and 3'-SAG2)) | 1 (1) | NO | - | - | - | - | 3 | 3 | [S27] |
|  |  |  |  | Slovakia | PCR-RFLP (SAG2 (5'-SAG2 and 3'-SAG2)) | 1 (1) | NO | - | 14 | - | - | - | 14 | [S28] |
|  |  | Cattle | *Bos taurus* | Italy | PCR-seq (SAG1, SAG2 (5'-SAG2), altSAG2, BTUB, c22-8 and GRA6) | 2 (1) | NO | 4 | 1 | 1 | - | - | 6 | [S29] |
|  |  |  |  | Portugal | PCR-RFLP (SAG1, SAG2 (5'-SAG2 and 3'-SAG2), altSAG2, SAG3, BTUB, c22-8, c29-2, GRA6, L358, PK1 and Apico) | 11 (11) | YES | 1 | - | - | - | - | 1 | [S30] |
|  |  |  |  | Portugal | PCR-Seq (SAG2 (5'-SAG2 and 3'-SAG2)) | 1 (1) | NO | - | 3 | - | - | - | 3 | [S27] |
|  |  |  |  | Switzerland | PCR-RFLP (SAG2 (5'-SAG2 and 3'-SAG2), altSAG2, SAG3, BTUB, c22-8, c29-2, GRA6, L358, PK1 and Apico) | 3 (1) | NO | 2 | - | 1 | 5 | 1 | 9 | [S31] |
|  |  |  |  | UK | PCR-RFLP and PCR-Seq (SAG2 (5'-SAG2 and 3'-SAG2) | 1 (1) | NO | - | - | - | 1 | - | 1 | [S32] |
|  |  | Domestic pig | *Sus scrofa dom.* | Czech Republic | PCR-RFLP (altSAG2, SAG3, BTUB, GRA6, L358, PK1 and Apico) | 7 (7) | NO | - | 17 | - | - | - | 17 | [S33] |
|  |  |  |  | France | PCR-RLFP (SAG1, SAG2 (5'-SAG2 and 3'-SAG2), altSAG2, SAG3, BTUB, GRA6, c22-8, c29-2, L358, PK1 and Apico) | 11 (11) | YES | - | 41 | - | - | - | 41 | [S34] |
|  |  |  |  | Italy | HRM and B1-seq | 1 (1) | NO | - | 2 | 4 | 1 | - | 7 | [S35] |
|  |  |  |  | Italy | PCR-Seq (B1) | 1 (1) | NO | 3 | 4 | 2 | - | - | 9 | [S36] |
|  |  |  |  | Italy | PCR-seq (altSAG2 and GRA6) | 2 (1) | NO | 14 | - | - | 1 | - | 15 | [S29] |
|  |  |  |  | Italy | HRM and B1-seq | 1 (1) | NO | 4 | 6 | 4 | - | - | 14 | [S37] |
|  |  |  |  | Poland | PCR-RFLP (SAG1, SAG2 (5'-SAG2 and 3'-SAG2), altSAG2, SAG3, BTUB, GRA6, c22-8, c29-2, L358, PK1 and Apico) | 10 (1) | NO | 4 | 9 | - | 1 | - | 14 | [S38] |
|  |  |  |  | Portugal | PCR-RFLP (SAG2 (5'-SAG2 and 3'-SAG2)) | 1 (1) | YES | - | 11 | 4 | - | - | 15 | [S39] |
|  |  |  |  | Portugal | PCR-Seq (SAG2 (5'-SAG2 and 3'-SAG2)) | 1 (1) | NO | - | 3 | - | - | 3 | 6 | [S27] |
|  |  |  |  | Serbia | PCR-RFLP (аltSAG2, BTUB, GRA6, c22-8, PK1, Apico and CS3) | 7 (3) | YES | - | 7 | 1 | 1 | - | 9 | [S40] |
|  |  |  |  | Slovakia | PCR-RFLP (SAG2 (5'-SAG2 and 3'-SAG2), altSAG2, ROP1) | 2 (2) | NO | 18 | 3 | - | - | - | 21 | [S41] |
| **Compartment** | **Order** | **Host** | **Species** | **Country** | **Molecular markers** | **Markers**  **Max (Min)** | **Isolation** | **Type I** | **Type II** | **Type III** | **MRA** | **ND** | **TOTAL** | **Reference** |
|  |  |  | *Sus scrofa dom.* | Spain | PCR-RFLP (SAG1, SAG2 (5'-SAG2 and 3'-SAG2), altSAG2, SAG3, BTUB, GRA6, c22-8, c29-2, L358, PK1, Apico and CS3) and PCR-Seq (CS3) | 12 (12) | YES | - | 3 | 2 | - | - | 5 | [S42] |
| Domestic animals |  | Domestic pig |  | Switzerland | PCR-RFLP (SAG2 (5'-SAG2 and 3'-SAG2), altSAG2, SAG3, BTUB, GRA6, c22-8, c29-2, L358, PK1 and Apico) | 3 (3) | NO | - | 1 | - | - | - | 1 | [S31] |
|  | Artiodactyla |  |  | UK | PCR-RFLP and PCR-Seq (SAG2 (5'-SAG2 and 3'-SAG2)) | 1 (1) | NO | 16 | - | - | 3 | - | 19 | [S32] |
|  |  | Sheep | *Ovis aries* | France | PCR-RFLP (SAG1, SAG2, GRA7) | 3 (3) | YES | - | 45 | 1 | - | - | 46 | [S43] |
|  |  |  |  | Ireland | PCR-RFLP (SAG2 (5'-SAG2 and 3'-SAG2), SAG3, BTUB and GRA6) | 4 (4) | NO | - | 15 | 4 | - | - | 19 | [S44] |
|  |  |  |  | Italy | PCR-RFLP (SAG3) | 1 (1) | NO | 1 | - | - | - | - | 1 | [S45] |
|  |  |  |  | Italy | PCR-Seq (B1) | 1 (1) | NO | - | 15 | - | - | - | 15 | [S24] |
|  |  |  |  | Italy | PCR-RFLP (SAG1, SAG2 (5'-SAG2 and 3'-SAG2), altSAG2, SAG3, BTUB, GRA6, c22-8, c29-2, L358, PK1 and Apico) | 11 (11) | YES | - | - | - | 5 | - | 5 | [S46] |
|  |  |  |  | Italy | PCR-RFLP (SAG1, SAG2 (5'-SAG2 and 3'-SAG2), SAG3, BTUB, GRA6, c22-8, c29-2, L358, PK1 and Apico) | 10 (10) | NO | - | 21 | - | - | - | 21 | [S47] |
|  |  |  |  | Portugal | PCR-Seq (SAG2 (5'-SAG2 and 3'-SAG2)) | 1 (1) | NO | - | 4 | - | - | 2 | 6 | [S27] |
|  |  |  |  | Serbia | PCR-RFLP (SAG1, SAG2 (5'-SAG2 and 3'-SAG2), GRA6, GRA7 (5`-GRA7 and 3`-GRA7)) | 4 (4) | YES | - | 1 | - | - | - | 1 | [S15] |
|  |  |  |  | Spain | PCR-RFLP (SAG1, SAG2 (5'-SAG2 and 3'-SAG2), altSAG2, SAG3, BTUB, GRA6, c22-8, c29-2, L358, PK1, Apico and CS3) and PCR-Seq (SAG3, GRA6, GRA7) | 12 (1) | YES (31), NO (144) | 4 | 164 | 2 | 4 | 1 | 175 | [S48] |
|  |  |  |  | Switwerland | PCR-RFLP (SAG2 (5'-SAG2 and 3'-SAG2), altSAG2, SAG3, BTUB, GRA6, c22-8, c29-2, L358, PK1 and Apico) | 10 (5) | NO | - | 2 | - | 3 | - | 5 | [S31] |
|  |  |  |  | The Netherlands | PCR-Seq (GRA6) | 1 (1) | NO | - | 16 | - | - | - | 16 | [S49] |
|  |  |  |  | UK | PCR-RFLP (SAG2 (5'-SAG2 and 3'-SAG2)) | 1 (1) | YES (2), NO (13) | - | 15 | - | - | - | 15 | [S50] |
|  |  |  |  | UK | PCR-RFLP and PCR-Seq (SAG2 (5'-SAG2 and 3'-SAG2) | 1 (1) | NO | 4 | - | - | 2 | - | 6 | [S32] |
|  | Carnivora | Cat | *Felis catus* | Italy | PCR-RFLP (SAG1, SAG2 (5'-SAG2 and 3'-SAG2), altSAG2, SAG3, BTUB, GRA6, c22-8, c29-2, L358, PK1 and Apico) | 8 (2) | NO | 7 | 1 | 7 | - | - | 15 | [S51] |
|  |  |  |  | Portugal | PCR-Seq (SAG2 (5'-SAG2 and 3'-SAG2)) | 1 (1) | YES | - | 71 | - | 1 | 10 | 82 | [S52] |
|  |  |  |  | Spain | PCR-RFLP (SAG2 (5'-SAG2 and 3'-SAG2)) | 1(1) | NO | 12 | 34 | - | - | 1 | 47 | [S53] |
|  |  |  |  | Switzerland | PCR-RFLP (altSAG2, SAG3, BTUB, GRA6, c22-8, c29-2, L358, PK1 and Apico) | 9 (9) | YES | - | 1 | - | - | - | 1 | [S54] |
| **Compartment** | **Order** | **Host** | **Species** | **Country** | **Molecular markers** | **Markers**  **Max (Min)** | **Isolation** | **Type I** | **Type II** | **Type III** | **MRA** | **ND** | **TOTAL** | **Reference** |
| Domestic animals | Carnivora | Cat | *Felis catus* | Switzerland | PCR-RFLP (SAG2 (5'-SAG2 and 3'-SAG2), altSAG2, SAG3, BTUB, GRA6, c22-8, c29-2, L358, PK1 and Apico) | 10(10) | NO | - | 1 | - | - | - | 1 | [S31] |
|  |  |  |  | Denmark | PCR-RFLP (SAG2 (5'-SAG2 and 3'-SAG2), altSAG2, SAG3, BTUB, GRA6, c22-8, c29-2, L358, PK1 and Apico) | 9 (9) | YES | - | 1 | - | - | - | 1 | [S55], [S56] |
|  |  |  |  | France |  | 9 (9) | YES | - | 2 | - | - | - | 2 |  |
|  |  |  |  | Germany |  | 10 (2) | YES | - | 63 | 1 | 4 | - | 68 |  |
|  |  |  |  | The Netherlands |  | 9 (9) | YES | - | 1 | - | - | - | 1 |  |
|  |  |  |  | Austria | PCR-RFLP (SAG2 (5'-SAG2 and 3'-SAG2), SAG3, BTUB and GRA6) | 4 (4) | NO | - | 1 | - | - |  | 1 | [S56] |
|  |  |  |  | France |  | 3 (3) | NO | - | 2 | - | - | - | 2 |  |
|  |  |  |  | Switzerland |  | 4 (4) | NO | - | 1 | - | - | - | 1 |  |
|  |  | Dog | *Canis lupus familiaris* | Germany | PCR-RFLP (altSAG2, SAG3, BTUB, GRA6, c22-8, c29-2, L358, PK1 and Apico) | 9 (9) | YES | - | 2 | - | - | - | 2 | [S55] |
|  |  |  |  | Italy |  | 9 (9) | YES | - | 1 | - | - | - | 1 |  |
|  |  |  |  | Italy | PCR-RFLP (SAG2 (5'-SAG2 and 3'-SAG2)) | 1 (1) | NO | 1 | - | - | - | - | 1 | [S57] |
|  | Galliformes | Chicken | *Gallus gallus dom.* | Italy | PCR-RFLP (SAG1, SAG2 (5'-SAG2 and 3'-SAG2), SAG3, BTUB, c22-8, c29-2, GRA6, L358, PK1, and Apico) | 10 (10) | YES | - | 3 | - | - | - | 3 | [S58] |
|  |  |  |  | Poland |  | 10 (10) | YES | - | - | - | 2 |  | 2 |  |
|  |  |  |  | Germany | PCR-RFLP (altSAG2, SAG3, BTUB, c22-8, c29-2, GRA6, L358 and PK1) | 8 (8) | YES | - | 15 | - | - | - | 15 | [S59] |
|  |  |  |  | Austria | PCR-RFLP (SAG1, SAG2 (5'-SAG2 and 3'-SAG2), altSAG2, SAG3, BTUB, GRA6, c22-8, c29-2, L358, PK1 and Apico) | 10 (8) | YES | - | 67 | - | - | - | 67 | [S30], [S60], [S61] |
|  |  |  |  | Portugal | PCR-RFLP (SAG1, SAG2 (5'-SAG2 and 3'-SAG2), altSAG2, SAG3, BTUB, c22-8, c29-2, GRA6, L358 and PK1) | 9 (9) | YES | - | 7 | 4 | 4 | - | 15 |  |
| Wild animals | Rodentia, Eulipotyphla | Micromammals | *Apodemus sylvaticus* | UK | PCR-RFLP (SAG2 (5'-SAG2 and 3'-SAG2), SAG3 and GRA6) | 3 (1) | NO | - | 8 | 1 | 35 | - | 44 | [S62] |
|  | Rodentia |  |  | UK | PCR-RFLP (SAG2 (5'-SAG2 and 3'-SAG2)) | 1 (1) | NO | - | 2 | - | - | - | 2 | [S50] |
|  |  |  | *Castor fiber* | Germany | PCR-RFLP (altSAG2, SAG3, BTUB, GRA6, c22-8, c29-2, L358, PK1 and Apico) | 9 (6) | NO | - | 2 | - | - | - | 2 | [S63] |
|  |  |  | *Rattus* sp. | Greece | PCR-RFLP (GRA6) | 1 (1) | NO | - | - | 2 | - | - | 2 | [S8] |
|  |  |  | *Rattus rattus* | Spain | PCR-RFLP (SAG1, SAG3, GRA6, c22-8 and Apico) | 5 (5) | NO | - | - | - | 1 | - | 1 | [S64] |
|  |  |  | Several species | Croatia | PCR-RFLP (SAG1, SAG2, GRA6 and GRA7) | 4 (4) | NO | - | 2 | - | - | - | 2 | [S65] |
|  |  |  | Several species | Poland | PCR-Seq (SAG1, SAG2 (5'-SAG2 and 3'-SAG2), SAG3, BTUB and GRA6) | 4 (1) | NO | - | 1 | 1 | - | 4 | 6 | [S66] |
|  |  |  | Several species | Slovakia | PCR-RFLP (SAG2 (5'-SAG2 and 3'-SAG2)) | 1 (1) | NO | 2 | - | - | - | - | 2 | [S67] |
| **Compartment** | **Order** | **Host** | **Species** | **Country** | **Molecular markers** | **Markers**  **Max (Min)** | **Isolation** | **Type I** | **Type II** | **Type III** | **MRA** | **ND** | **TOTAL** | **Reference** |
|  | Cetacea | Marine mammals | *Stenella coeruleoalba* | Italy | PCR-Seq (GRA6) | 1 (1) | NO | - | 3 | - | - | - | 3 | [S68] |
|  |  |  |  | Italy | PCR-RFLP (SAG1, SAG2 (5'-SAG2 and 3'-SAG2), altSAG2, SAG3, BTUB and GRA6) | 6 (6) | NO | - | 1 | - | - | - | 1 | [S69] |
|  |  |  |  | Italy | PCR-RFLP (SAG1, SAG2 (5'-SAG2 and 3'-SAG2), altSAG2, SAG3, BTUB, GRA6, c22-8, c29-2, L358, PK1 and Apico) | 11 (11) | NO | - | 1 | - | - | - | 1 | [S70] |
| Wild animals |  |  | *Phocoena phocoena* | Denmark | PCR-RFLP (altSAG2, BTUB, GRA6, SAG3, c22-8, c29-2, L358, PK1 and Apico) | 9 (9) | NO | - | 1 | - | - | - | 1 | [S71] |
|  | Carnivora | Mesocarnivores | *Vulpes lagopus* | Norway | PCR-RFLP (SAG1, altSAG2, SAG3, BTUB, GRA6, c22-8, c29-2, L358, PK1 and Apico) and PCR-Seq (BSR4, GRA6, UPRT1 and UPRT2) | 13 (13) | YES | - | 1 | - | - | - | 1 | [S72] |
|  |  |  |  | Norway | PCR-RFLP (SAG1, SAG2 (5'-SAG2 and 3'-SAG2), altSAG2, SAG3, BTUB, GRA6, L358, c22-8, c29-2, PK1 and Apico) | 11 (4) | NO | - | 46 | 7 | 2 | - | 55 | [S73] |
|  |  |  | *Vulpes vulpes* | Germany | PCR-RFLP (altSAG2, SAG3, BTUB, GRA6, c22-8, c29-2, L358, PK1 and Apico) | 9 (2) | NO | - | 37 | 1 | 2 | - | 40 | [S74] |
|  |  |  |  | Italy | PCR-Seq (SAG1, SAG2 (5'-SAG2), altSAG2, GRA6 and c22-8) | 5 (1) | NO | 5 | - | - | 2 | - | 7 | [S29] |
|  |  |  |  | Italy | PCR-RFLP (SAG1, SAG2 (5'-SAG2 and 3'-SAG2), SAG3, altSAG2, BTUB, GRA6, c22-8, c29-2, L358, PK1 and Apico) and PCR-Seq (SAG3) | 9 (2) | NO | 1 | - | - | 7 | - | 8 | [S75] |
|  |  |  | Several species | Poland | PCR-RFLP (B1) and PCR-Seq (SAG1, SAG2 (5'-SAG2 and 3'-SAG2), SAG3, BTUB and GRA6) | 3 (1) | NO | - | 2 | 1 | 2 | 2 | 7 | [S66] |
|  |  |  | Several species | Serbia | PCR-RFLP (altSAG2, BTUB, GRA6, c22-8, c29-2, L358, PK1, Apico and CS3) | 7 (4) | NO | - | 4 | 3 | 16 | - | 23 | [S76] |
|  |  |  | Several species | Slovakia | PCR-RFLP (SAG2 (5'-SAG2 and 3'-SAG2)) | 1 (1) | NO | - | 5 | 5 | - | - | 10 | [S67] |
|  |  |  | Several species | UK | PCR-RFLP (SAG2 (5'-SAG2 and 3'-SAG2), SAG3, BTUB and GRA6) and PCR-Seq (SAG2 (5'-SAG2 and 3'-SAG2), SAG3, BTUB and GRA6) | 4 (1) | NO | 2 | 29 | 1 | - | - | 32 | [S77] |
|  |  | Wild cat | *Felis sylvestris* | Germany | PCR-RFLP (altSAG2, SAG3, BTUB, GRA6, c22-8, c29-2, L358, PK1 and Apico) | 9 (6) | NO | - | 3 | - | - | - | 3 | [S63] |
|  |  |  |  | Slovakia | PCR-RFLP (SAG2 (5'-SAG2 and 3'-SAG2)) | 1(1) | NO | - | - | 1 | - | - | 1 | [S67] |
|  |  |  | *Lynx lynx* |  |  | 1(1) | NO | 1 | - | - | - | - | 1 |  |
|  | Artiodactyla | Wild ungulates | *Bison bonasus bonasus* | Poland | PCR-RFLP (SAG1, SAG2 (5'-SAG2 and 3'-SAG2), altSAG2, SAG3, BTUB, GRA6, c22-8, c29-2, L358, PK1 and Apico) | 11 (11) | YES | 1 | - | - | - | - | 1 | [S78] |
|  |  |  | *Capreolus capreolus* | Italy | PCR-Seq (altSAG2 and GRA6) | 2 (2) | NO | 1 | - | - | - | - | 1 | [S29] |
|  |  |  | *Rupicapra rupicapra* | Italy | PCR-RFLP (SAG2 (5'-SAG2 and 3'-SAG2)) | 1 (1) | NO | - | 1 | - | - | - | 1 | [S79] |
|  |  |  | Cervidae (venison) | UK | PCR-RFLP (SAG2 (5'-SAG2 and 3'-SAG2), altSAG2, SAG3, BTUB, GRA6, c22-8, L358 and Apico) | 4 (3) | NO | 1 | 1 | - | 1 | - | 3 | [S80] |
|  |  |  | *Cervus elaphus* | Italy | PCR-RFLP (SAG1, SAG2 (5'-SAG2 and 3'-SAG2), altSAG2, SAG3, BTUB, GRA6, c22-8, c29-2, L358 and PK1) | 10 (10) | NO | - | 1 | - | - | - | 1 | [S81] |
| **Compartment** | **Order** | **Host** | **Species** | **Country** | **Molecular markers** | **Markers**  **Max (Min)** | **Isolation** | **Type I** | **Type II** | **Type III** | **MRA** | **ND** | **TOTAL** | **Reference** |
| Wild animals | Artiodactyla | Wild ungulates | Several species | France | PCR-RFLP (SAG1, SAG2 and GRA7) | 3 (3) | YES | - | 12 | - | - | - | 12 | [S82] |
|  |  |  | Several species | Spain | PCR-RFLP (SAG1, SAG3, BTUB and GRA6) | 2 (1) | NO | - | 13 | 2 | 1 | - | 16 | [S83] |
|  |  | Wild pig | *Sus scrofa ferus* | Czech Republic | PCR-RFLP (altSAG2, SAG3, BTUB, GRA6, L358, PK1 and Apico) | 7 (7) | NO | - | 38 | - | - | - | 38 | [S33] |
|  |  |  |  | France | PCR-RFLP (SAG1, SAG2, GRA7) | 3 (3) | YES | - | 21 | - | - | - | 21 | [S84] |
|  |  |  |  | Italy | PCR-RFLP (SAG1, SAG2 (5'-SAG2 and 3'-SAG2), altSAG2, SAG3, BTUB, GRA6, c22-8, c29-2, L358, PK1, Apico) | 11 (4) | NO | - | 2 | - | 9 | - | 11 | [S85] |
|  |  |  |  | Italy | PCR-Seq (SAG1, SAG2 (5'-SAG2), altSAG2, BTUB, GRA6, and c22-8) | 6 (1) | NO | 7 | 1 | 2 | 4 | - | 14 | [S29] |
|  |  |  |  | Italy | PCR-seq (B1, BTUB and GRA6) | 3 (1) | NO | - | 2 | - | - | - | 2 | [S86] |
|  |  |  |  | Spain | PCR-RFLP (SAG1, SAG2 (5'-SAG2 and 3'-SAG2), SAG3, BTUB and GRA6) | 4 (1) | NO | 2 | 2 | - | 3 | - | 7 | [S83] |
|  |  |  |  | Spain | PCR-RFLP (SAG1, SAG2 (5'-SAG2 and 3'-SAG2), SAG3, BTUB and GRA6) | 5 (2) | NO | 1 | - | 1 | 2 | - | 4 | [S87] |
|  | Passeriformes | Wild avian spp. | *Pica* spp. | Italy | PCR-RFLP (SAG1, SAG2 (5'-SAG2 and 3'-SAG2), altSAG2 and SAG3) | 4 (4) | NO | - | 8 | 7 | - | - | 15 | [S88] |
|  | Columbiformes |  | *Columba* spp. | Portugal | PCR-Seq (SAG2 (5'-SAG2 and 3'-SAG2)) | 1 (1) | YES | 1 | 25 | 2 | 1 | - | 29 | [S52] |
|  |  |  |  | Serbia | PCR-RFLP (SAG1, SAG2 (5'-SAG2 and 3'-SAG2), GRA6 and GRA7 (5’-GRA7 and 3’-GRA7)) | 4 (4) | YES | - | 2 | 1 | - | - | 3 | [S15] |
|  | Anseriformes |  | *Anas* spp. | France | PCR-RFLP (SAG1, SAG2 and GRA7) | 3 (3) | YES | - | 1 | - | - | - | 1 | [S82] |
|  |  |  |  | Italy | PCR-RFLP (SAG1, SAG2 (5'-SAG2 and 3'-SAG2), altSAG2, SAG3, BTUB, GRA6, c22-8, c29-2, L358, PK1 and Apico) | 11 (8) | NO | - | - | - | 3 | - | 3 | [S89] |
|  | Anseriformes, Galliformes |  | Anas spp., *Phasianus* spp. | Czech Republic | PCR-RFLP (SAG3, BTUB, GRA6, L358, PK1 and Apico) | 6 (3) | NO | - | 7 | 11 | 10 | - | 28 | [S90] |
|  | Strigiformes, Accipitriformes, Passeriformes |  | Several species | Slovakia | PCR-RFLP (SAG2 (5'-SAG2 and 3'-SAG2)) | 1 (1) | NO | - | 2 | 2 | - | - | 4 | [S67] |
|  | Falconiformes, Charadriiformes, Accipitriformes |  | Several species | Poland | PCR-RFLP (B1) | 1 (1) | NO | - | - | - | - | 3 | 3 | [S66] |
| Environment | - | - | Fresh produce | Czech Republic | PCR-RFLP (SAG3, BTUB, GRA6, c22-8, c29-2, L358 and PK1) | 7 (7) | NO | - | 7 | - | - | - | 7 | [S91] |
|  |  |  |  | Poland | PCR-RFLP (SAG2 (5'-SAG2 and 3'-SAG2)) | 1 (1) | NO | 6 | 2 | - | - | - | 8 | [S92] |
|  |  |  |  | Italy | PCR-Seq (B1) | 1 (1) | NO | 5 | - | - | - | - | 5 | [S93] |
|  |  |  | Soil | Poland | PCR-RFLP (SAG2 (5'-SAG2 and 3'-SAG2)) | 1 (1) | NO | 5 | 2 | - | - | - | 7 | [S94] |
| **Compartment** | **Order** | **Host** | **Species** | **Country** | **Molecular markers** | **Markers**  **Max (Min)** | **Isolation** | **Type I** | **Type II** | **Type III** | **MRA** | **ND** | **TOTAL** | **Reference** |
| Environment | - | - | Water | Poland | PCR-Seq (B1) | 1 (1) | NO | 7 | - | - | - | - | 7 | [S95] |
|  |  |  |  | Poland | PCR-RFLP (B1) | 1 (1) | YES(9)/NO(26) | 32 | - | - | - | 3 | 35 | [S96] |
|  |  |  | Air | Poland | PCR-RFLP (SAG2 (5'-SAG2 and 3'-SAG2)) | 1 (1) | NO | 2 | - | - | - | - | 2 | [S97] |
|  | Ixodida | Ticks | Ixodid tick | Poland | PCR-RFLP (B1, SAG1, SAG2 (5'-SAG2 and 3'-SAG2), SAG3 and GRA6) | 5 (1) | NO | 21 | - | - | - | - | 21 | [S98] |
|  |  |  |  |  | PCR-Seq (B1) | 1 (1) | NO | 12 | - | - | - | - | 12 | [S99] |
|  |  |  |  |  | PCR-RFLP (B1) | 1 (1) | NO | 26 | - | - | 5 | 2 | 33 | [S100] |
|  |  |  |  |  | PCR-RFLP (B1) | 1 (1) | NO | 41 | - | - | 41 | 8 | 90 | [S101] |
| **TOTAL** |  |  |  |  |  |  |  | **390** | **1268** | **194** | **220** | **55** | **2127** |  |

**Table S2.** Data extracted from available studies on European *Toxoplasma gondii* strains microsatellites genotyping deposited in PubMed database (n=43). Typing results on both, isolated viable parasites and DNA positive specimens/clinical samples have been considered; data from overseas territories and zoo-kept animals were not included.

| **Compartment** | **Order** | **Host** | **Species** | **Country** | **Microsatellite markers** | **Markers**  **Max (Min)** | **Isolation** | **Type I** | **Type II** | **Type III** | **NonCan-Mixed** | **Africa1** | **Caribbean2** | **Caribbean3** | **ND** | **TOTAL** | **Reference** |
| --- | --- | --- | --- | --- | --- | --- | --- | --- | --- | --- | --- | --- | --- | --- | --- | --- | --- |
| Human | Primates | Human | *Homo sapiens* | Austria | TUB2, TgM-A, W35, B17, B18, M33 | 6 (6) | YES | - | 1 | - | - | - | - | - | - | 1 | [S102] |
|  |  |  |  | Belgium | TUB2, TgM-A, W35, B17, B18, M33//IV.1, XI.1, M48, M102, N60, N82, AA, N61, N83 | 15 (15) | YES | - | - | - | - | 1 | - | - | - | 1 | [S103] |
|  |  |  |  | Belgium | TUB2, TgM-A, W35, B17, B18, M33//IV.1, XI.1, M48, M102, N60, N82, AA, N61, N83 | 15 (14) | NO | - | 14 | - | - | - | - | - | - | 14 | [S104] |
|  |  |  |  | Belgium | TUB2, TgM-A, W35, B17, B18, M33//IV.1, XI.1, M48, M102, N60, N82, AA, N61, N83 | 15 (15) | YES | - | 4 | - | - | - | - | - | - | 4 | [S105] |
|  |  |  |  | Denmark | TUB2, TgM-A, W35, B17, B18, M33//IV.1, XI.1, M48, M102, N60, N82, AA, N61, N83 | 15 (3) | NO | - | 14 | 2 | 2 | 2 | 1 | - | - | 21 | [S106] |
|  |  |  |  | England | TUB2, TgM-A, W35, B17, B18, M33//IV.1, XI.1, M48, M102, N60, N82, AA, N61, N83 | 15 (15) | YES | - | 1 | - | - | - | - | - | - | 1 | [S107] |
|  |  |  |  | France | TUB2, TgM-A, W35, B17, B18, M33//IV.1, XI.1, M48, M102, N60, N82, AA, N61, N83 | 15 (15) | YES | 2 | - | 1 | 2 | 1 | - | - | - | 6 | [S103] |
|  |  |  |  | France | TUB2, TgM-A, W35, B17, B18 | 5 (5) | YES | 1 | 1 | 3 | 1 | - | - | - | - | 6 | [S108] |
|  |  |  |  | France | TUB2, TgM-A, W35, B17, B18, M34 | 6 (6) | YES (11)/NO (34) | - | 39 | 4 | 2 | - | - | - | - | 45 | [S102] |
|  |  |  |  | France | TUB2, TgM-A, W35, B17, B18, M33//IV.1, XI.1, M48, M102, N60, N82, AA, N61, N83 | 15 (15) | YES | - | 240 | - | - | - | - | - | - | 240 | [S109] |
|  |  |  |  | France | TUB2, TgM-A, W35, B17, B18, M33//IV.1, XI.1, M48, M102, N60, N82, AA, N61, N83 | 15 (15) | YES | - | 1 | - | - | - | - | - | - | 1 | [S110] |
|  |  |  |  | France | TUB2, TgM-A, W35, B17, B18 | 5 (5) | NO | - | - | - | 1 | - | - | - | - | 1 | [S111] |
|  |  |  |  | France | TUB2, TgM-A, W35, B17, B18 | 5 (5) | NO | - | - | - | 2 | - | - | - | - | 2 | [S112] |
|  |  |  |  | France | TUB2, TgM-A, W35, B17, B18 | 5 (1) | NO | - | 10 | - | 1 | 2 | - | - | 7 | 20 | [S113] |
|  |  |  |  | France | TUB2, TgM-A, W35, B17, B18 | 5 (5) | YES | - | - | - | 1 | - | - | - | - | 1 | [S114] |
|  |  |  |  | France | TUB2, TgM-A, W35, B17, B18, M33//IV.1, XI.1, M48, M102, N60, N82, AA, N61, N83 | 15 (15) | YES | - | - | - | 1 | - | - | - | - | 1 | [S115] |
|  |  |  |  | France | TUB2, TgM-A, W35, B17, B18, M33//IV.1, XI.1, M48, M102, N60, N82, AA, N61, N83 | 15 (15) | YES | - | 2 | 3 | - | - | - | - | - | 5 | [S116] |
| **Compartment** | **Order** | **Host** | **Species** | **Country** | **Microsatellite markers** | **Markers**  **Max (Min)** | **Isolation** | **Type I** | **Type II** | **Type III** | **NonCan-Mixed** | **Africa1** | **Caribbean2** | **Caribbean3** | **NA** | **TOTAL** | **Reference** |
| Human | Primates | Human | *Homo sapiens* | France | TUB2, TgM-A, W35, B17, B18, M33//IV.1, XI.1, M48, M102, N60, N82, AA, N61, N83 | 15 (15) | NO | - | 1 | - | - | - | - | - | - | 1 | [S117] |
|  |  |  |  | France | TUB2, TgM-A, W35, B17, B18, M33//IV.1, XI.1, M48, M102, N60, N82, AA, N61, N83 | 15 (15) | YES | 1 | 1 | - | - | - | - | - | - | 2 | [S107] |
|  |  |  |  | France | TUB2, TgM-A, W35, B17, B18, M33//IV.1, XI.1, M48, M102, N60, N82, AA, N61, N83 | 15 (15) | YES | - | 8 | - | - | - | - | - | - | 8 | [S105] |
|  |  |  |  | France | TUB2, TgM-A, W35, B17, B18, M33//IV.1, XI.1, M48, M102, N60, N82, AA, N61, N83 | 15 (15) | YES | - | - | - | 2 | 1 | - | - | - | 3 | [S118] |
|  |  |  |  | France-imported | TUB2, TgM-A, W35, B17, B18 | 5 (5) | NO | - | - | - | 1 | - | - | - | - | 1 | [S119] |
|  |  |  |  | Germany | TUB2, TgM-A, W35, B17, B18, M33//IV.1, XI.1, M48, M102, N60, N82, AA, N61, N83 | 15 (15) | YES | - | 1 | - | - | - | - | - | - | 1 | [S103] |
|  |  |  |  | Germany | TUB2, TgM-A, W35, B17, B18, M33//IV.1, XI.1, M48, M102, N60, N82, AA, N61, N83 | 15 (15) | YES | - | 2 | - | - | - | - | - | - | 2 | [S105] |
|  |  |  |  | Portugal | TUB2, TgM-A, W35, B17, B18, M35 | 6 (6) | NO | - | 2 | - | - | - | - | - | - | 2 | [S102] |
|  |  |  |  | Portugal | TUB2, TgM-A, W35, B17, B18 | 5 (5) | YES | 6 | 32 | - | 10 | - | - | - | - | 48 | [S13] |
|  |  |  |  | Romania | TUB2, TgM-A, W35, B17, B18, M33//IV.1, XI.1, M48, M102, N60, N82, AA, N61, N83 | 15 (15) | YES | - | 1 | - | - | - | - | - | - | 1 | [S110] |
|  |  |  |  | Serbia | TUB2, TgM-A, W35, B17, B18, M33//IV.1, XI.1, M48, M102, N60, N82, AA, N61, N83 | 14 (14) | YES | - | - | - | 1 | - | - | - | - | 1 | [S16] |
|  |  |  |  | The Netherlands | TUB2, TgM-A, W35, B17, B18, M33//IV.1, XI.1, M48, M102, N60, N82, AA, N61, N83 | 15 (15) | YES | 1 | - | - | - | - | - | - | - | 1 | [S103] |
| Domestic  animals | Artiodactyla | Cattle | *Bos taurus* | France | TUB2, TgM-A, W35, B17, B18, M33//IV.1, XI.1, M48, M102, N60, N82, AA, N61, N83 | 15 (15) | YES | - | 2 | - | - | - | - | - | - | 2 | [S120] |
|  |  |  |  | Portugal | TUB2, TgM-A, W35, B17, B18, M33//IV.1, XI.1, M48, M102, N60, N82, AA, N61, N83 | 15 (15) | YES | 1 | - | - | - | - | - | - | - | 1 | [S107] |
|  |  |  |  | Portugal | TUB2, TgM-A, W35, B17, B18, M33//IV.1, XI.1, M48, M102, N60, N82, AA, N61, N83 | 15 (15) | YES | 1 | - | - | - | - | - | - | - | 1 | [S30] |
|  |  | Goat | *Capra aegagrus hircus* | Romania | TUB2, TgM-A, W35, B17, B18, M33//IV.1, XI.1, M48, M102, N60, N82, AA, N61, N83 | 15 (15) | YES | - | 2 | - | - | - | - | - | - | 2 | [S121] |
|  |  | Domestic pig | *Sus scrofa domesticus* | Portugal | TUB2, TgM-A, W35, B17, B18, M33//IV.1, XI.1, M48, M102, N60, N82, AA, N61, N83 | 15 (15) | YES | - | 4 | 2 | - | - | - | - | - | 6 | [S122] |
|  |  |  |  | Portugal | TUB2, TgM-A, W35, B17, B18, M33//IV.1, XI.1, M48, M102, N60, N82, AA, N61, N83 | 15 (15) | YES | - | - | 1 | - | - | - | - | - | 1 | [S116] |
|  |  |  |  | Portugal | TUB2, TgM-A, W35, B17, B18 | 5 (5) | YES | - | 6 | 1 | 1 | - | - | - | - | 8 | [S39] |
|  |  |  |  | Romania | TUB2, TgM-A, W35, B17, B18, M33//IV.1, XI.1, M48, M102, N60, N82, AA, N61, N83 | 15 (15) | YES | - | 3 | - | - | - | - | - | - | 3 | [S121] |
| **Compartment** | **Order** | **Host** | **Species** | **Country** | **Microsatellite markers** | **Markers**  **Max (Min)** | **Isolation** | **Type I** | **Type II** | **Type III** | **NonCan-Mixed** | **Africa1** | **Caribbean2** | **Caribbean3** | **NA** | **TOTAL** | **Reference** |
| Domestic  animals | Artiodactyla | Sheep | *Ovis aries* | France | TUB2, TgM-A, W35, B17, B18, M33//IV.1, XI.1, M48, M102, N60, N82, AA, N61, N83 | 15 (14) | YES | - | 11 | 1 | - | - | - | - | - | 12 | [S122] |
|  |  |  |  | France | TUB2, TgM-A, W35, B17, B18, M33//IV.1, XI.1, M48, M102, N60, N82, AA, N61, N83 | 15 (15) | YES | - | 1 | - | - | - | - | - | - | 1 | [S110] |
|  |  |  |  | France | TUB2, TgM-A, W35, B17, B18 | 5 (5) | YES | - | 8 | - | - | - | - | - | - | 8 | [S123] |
|  |  |  |  | France | TUB2, TgM-A, W35, B17, B18, M33//IV.1, XI.1, M48, M102, N60, N82, AA, N61, N83 | 15 (15) | YES | - | 1 | 1 | - | - | - | - | - | 2 | [S116] |
|  |  |  |  | France | TUB2, TgM-A, W35, B17, B18, M33//IV.1, XI.1, M48, M102, N60, N82, AA, N61, N83 | 15 (15) | NO | - | 29 | - | - | - | - | - | - | 29 | [S105] |
|  |  |  |  | France | TUB2, TgM-A, W35, B17, B18, M33 | 6 (6) | YES | - | 34 | - | - | - | - | - | - | 34 | [S43] |
|  |  |  |  | The Netherlands | TUB2, TgM-A, W35, B17, B18, M33 | 6 (2) | NO | - | 7 | - | - | - | - | - | - | 7 | [S49] |
|  | Carnivora | Cat | *Felis catus* | Finland | TUB2, TgM-A, W35, B17, B18, M33//M48 | 7 (7) | YES (2)/NO(4) | - | 6 | - | - | - | - | - | - | 6 | [S124] |
|  |  |  |  | Germany | TUB2, TgM-A, W35, B17, B18, M33//IV.1, XI.1, M48, M102, N60, N82, AA, N61, N83 | 15 (15) | YES | - | 4 | - | - | - | - | - | - | 4 | [S105] |
|  |  |  |  | Portugal | TUB2, TgM-A, W35, B17, B18 | 5 (5) | YES | 1 | 16 | - | - | - | - | - | - | 17 | [S52] |
|  |  | Dog | *Canis lupus familiaris* | Italy | TUB2, TgM-A, W35, B17, B18, M33//IV.1, XI.1, M48, N82, AA, N61, N83 | 12 (12) | NO | 1 | - | - | - | - | - | - | - | 1 | [S57] |
|  | Galliformes | Chicken | *Gallus gallus dom.* | Austria | TUB2, TgM-A, W35, B17, B18, M33//IV.1, XI.1, M48, M102, N60, N82, AA, N61, N83 | 15 (9) | YES | - | 65 | - | - | - | - | - | - | 65 | [S30] |
|  |  |  |  | Germany | TUB2, TgM-A, W35, B17, B18, M33//IV.1, XI.1, M48, M102, N60, N82, AA, N61, N83 | 15 (15) | YES | - | 13 | - | - | - | - | - | - | 13 | [S105] |
|  |  |  |  | Portugal | TUB2, TgM-A, W35, B17, B18, M33//IV.1, XI.1, M48, M102, N60, N82, AA, N61, N83 | 15 (15) | YES | - | 7 | 8 | - | - | - | - | - | 15 | [S30] |
|  | Perissodactyla | Horse | *Equus ferus caballus* | Serbia | TUB2, TgM-A, W35, B17, B18, M33//IV.1, XI.1, M48, M102, N60, N82, AA, N61, N83 | 15 (15) | YES | - | - | 2 | - | - | - | - | - | 2 | [S116] |
| Wildlife | Cetacea | Marine mammals | *Balaenoptera physalus* | Italy | TUB2, TgM-A, W35, B17, B18, M33//IV.1, XI.1, M48, M102, N60, N82, AA, N61, N83 | 3 (3) | NO | - | 1 | - | - | - | - | - | - | 1 | [S125] |
|  | Artiodactyla | Wild Boar | *Sus scrofa ferus* | Belgium | TUB2, TgM-A, W35, B17, B18, M33//IV.1, XI.1, M48, M102, N60, N82, AA, N61, N83 | 15 (13) | YES | - | 9 | - | - | - | - | - | - | 9 | [S104] |
|  |  |  |  | France | TUB2, TgM-A, W35, B17, B18, M33//IV.1, XI.1, M48, M102, N60, N82, AA, N61, N83 | 15 (15) | NO | - | 1 | - | - | - | - | - | - | 1 | [S117] |
|  |  |  |  | France | TUB2, TgM-A, W35, B17, B18, M33 | 6 (6) | YES | - | 21 | - | - | - | - | - | - | 21 | [S84] |
|  |  |  |  | France | TUB2, TgM-A, W35, B17, B18, M33//IV.1, XI.1, M48, M102, N60, N82, AA, N61, N83 | 15 (15) | NO | - | 2 | - | - | - | - | - | - | 2 | [S105] |
|  |  |  |  | Italy | TUB2, TgM-A, W35, B17, B18 | 5 (5) | NO | - | 1 | 1 | 8 | - | - | 1 | - | 11 | [S126] |
| **Compartment** | **Order** | **Host** | **Species** | **Country** | **Microsatellite markers** | **Markers**  **Max (Min)** | **Isolation** | **Type I** | **Type II** | **Type III** | **NonCan-Mixed** | **Africa1** | **Caribbean2** | **Caribbean3** | **NA** | **TOTAL** | **Reference** |
| Wildlife | Artiodactyla | Wild ungulates | *Ovis gmelini* | France | TUB2, TgM-A, W35, B17, B18, M35 | 6 (6) | YES | - | 1 | - | - | - | - | - | - | 1 | [S82] |
|  |  |  | *Cervus elaphus* | France | TUB2, TgM-A, W35, B17, B18, M36 | 6 (6) | YES | - | 1 | - | - | - | - | - | - | 1 | [S82] |
|  |  |  | *Capreolus capreolus* | France | TUB2, TgM-A, W35, B17, B18, M37 | 6 (6) | YES | - | 12 | - | - | - | - | - | - | 12 | [S82] |
|  |  |  |  | France | TUB2, TgM-A, W35, B17, B18, M33//IV.1, XI.1, M48, M102, N60, N82, AA, N61, N83 | 15 (15) | NO | - | 1 | - | - | - | - | - | - | 1 | [S105] |
|  | Anseriformes | Wild avian spp. | *Anas platyrhynchos* | France | TUB2, TgM-A, W35, B17, B18, M34 | 6 (6) | YES | - | 1 | - | - | - | - | - | - | 1 | [S82] |
|  | Charadriiformes |  | *Larus michahellis* | Spain | TUB2, TgM-A, W35, B17, B18, M33//IV.1, XI.1, M48, M102, N60, N82, AA, N61, N83 | 15 (15) | YES | - | 1 | - | - | - | - | - | - | 1 | [S127] |
|  | Columbiformes |  | *Columba livia* | Portugal | TUB2, TgM-A, W35, B17, B18 | 5 (5) | YES | 1 | 12 | 2 | 1 | - | - | - | - | 16 | [S52] |
|  |  |  |  | Serbia | TUB2, TgM-A, W35, B17, B18, M33//IV.1, XI.1, M48, M102, N60, N82, AA, N61, N83 | 15 (15) | YES | - | - | 1 | - | - | - | - | - | 1 | [S116] |
|  | Carnivora | Mesocarnivores | *Vulpes vulpes* | Belgium | TUB2, TgM-A, W35, B17, B18, M33//IV.1, XI.1, M48, M102, N60, N82, AA, N61, N83 | 15 (15) | NO | - | 25 | 1 | - | - | - | - | - | 26 | [S128] |
|  |  |  |  | Belgium | TUB2, TgM-A, W35, B17, B18, M33//IV.1, XI.1, M48, M102, N60, N82, AA, N61, N83 | 15 (15) | NO | - | - | 1 | - | - | - | - | - | 1 | [S116] |
|  |  |  |  | Belgium | TUB2, TgM-A, W35, B17, B18, M33//IV.1, XI.1, M48, M102, N60, N82, AA, N61, N83 | 15 (15) | NO | - | 5 | - | - | - | - | - | - | 5 | [S105] |
|  |  |  |  | Czech Republic | TUB2, TgM-A, W35, B17, B18, M33//IV.1, XI.1, M48, M102, N60, N82, AA, N61, N83 | 15 (5) | NO | - | 5 | - | - | - | - | - | - | 5 | [S129] |
|  |  |  |  | France | TUB2, TgM-A, W35, B17, B18, M33 | 6 (6) | YES | - | 9 | - | - | - | - | - | - | 9 | [S82] |
|  |  |  |  | France | TUB2, TgM-A, W35, B17, B18, M33//IV.1, XI.1, M48, M102, N60, N82, AA, N61, N83 | 15 (15) | NO | - | 2 | - | - | - | - | - | - | 2 | [S105] |
|  |  |  |  | Norway | TUB2, TgM-A, W35, B17, B18, M33//IV.1, XI.1, M48, M102, N60, N82, AA, N61, N83 | 15 (14) | NO | - | 6 | - | - | - | - | - | - | 6 | [S105] |
|  |  |  | *Meles meles* | France | TUB2, TgM-A, W35, B17, B18, M33//IV.1, XI.1, M48, M102, N60, N82, AA, N61, N83 | 15 (15) | YES | - | - | - | 1 | - | - | - | - | 1 | [S116] |
|  | Lagomorpha | Hare | *Lepus europaeus* | Czech Republic | TUB2, TgM-A, W35, B17, B18, M33//IV.1, XI.1, M48, M102, N60, N82, AA, N61, N83 | 15 (13) | NO | - | 2 | - | - | - | - | - | - | 2 | [S130] |
|  |  |  |  | Finland | TUB2, TgM-A, W35, B17, B18, M33, M48 | 7 (5) | NO | - | 14 | - | - | - | - | - | - | 14 | [S131] |
|  |  |  | *Lepus timidus* | Finland | TUB2, TgM-A, W35, B17, B18, M33, M49 | 7 (7) | NO | - | 4 | - | - | - | - | - | - | 4 | [S131] |
|  |  | Rabbit | *Oryctolagus cuniculus* | England | TUB2, TgM-A, W35, B17, B18, M33//IV.1, XI.1, M48, M102, N60, N82, AA, N61, N83 | 14 (14) | YES | - | 1 | - | - | - | - | - | - | 1 | [S105] |
|  | Rodentia | Micro  mammals | *Sciurus vulgaris* | Finland | TUB2, TgM-A, W35, B17, B18, M33//M48 | 7 (7) | NO | - | 3 | - | - | - | - | - | - | 3 | [S132] |
| **Compartment** | **Order** | **Host** | **Species** | **Country** | **Microsatellite markers** | **Markers**  **Max (Min)** | **Isolation** | **Type I** | **Type II** | **Type III** | **NonCan-Mixed** | **Africa1** | **Caribbean2** | **Caribbean3** | **NA** | **TOTAL** | **Reference** |
| Wildlife | Rodentia | Micro  mammals | *Apodemus flavicollis* | Czech  Republic | TUB2, TgM-A, W35, B17, B18, M33//IV.1, XI.1, M48, M102, N60, N82, AA, N61, N83 | 15 (15) | NO | - | 2 | - | - | - | - | - | - | 2 | [S133] |
|  |  |  | *Apodemus sylvestris* | Czech Republic | TUB2, TgM-A, W35, B17, B18, M33//IV.1, XI.1, M48, M102, N60, N82, AA, N61, N83 | 15 (15) | NO | - | 1 | - | - | - | - | - | - | 1 | [S133] |
| Environment | Mytilida | Bivalve molluscs | *Mytilus galloprovincialis* | Italy | TUB2, TgM-A, W35, B17, B18 | 5 (5) | NO | 4 | - | - | 1 | - | - | - | - | 5 | [S134] |
| **TOTAL** |  |  |  |  |  |  |  | **20** | **737** | **35** | **39** | **7** | **1** | **1** | **7** | **847** |  |

**Table S3.** Sequence data available at GenBank for European *Toxoplasma gondii* isolates and samples.

| **GenBank#** | **Marker or gene** | **Isolate ID** | **Country** | **Animals** | **Sample type** | **Comment** | **First author and year** |
| --- | --- | --- | --- | --- | --- | --- | --- |
| MN958069 | GRA6 | ID60322 | Italy | Otter | Tissue | Included into analysis | [S135] |
| MT321285 | GRA6 | ME49-like | Italy | Goat | Tissue | Included into analysis | [S25] |
| MT370491 | GRA6 | ID_18-15_2 | Spain | Sheep | Fetal tissue | Included into analysis | [S48] |
| MT370490 | GRA6 | TgShSp24 | Spain | Sheep | Tissue | Included into analysis |  |
| MT370489 | GRA6 | ID_18-14_5 | Spain | Sheep | Fetal tissue | Included into analysis |  |
| MH429071 | GRA6 | 73_Gra6__W_III | Poland | NA | Raw meat product | Included into analysis | [S136] |
| MH429070 | GRA6 | 62_Gra6__W_III | Poland | NA | Raw meat product | Included into analysis |  |
| MH429069 | GRA6 | 34_Gra6__j_III | Poland | NA | Raw meat product | Included into analysis |  |
| MH429068 | GRA6 | 79_Gra6__A_III | Poland | NA | Raw meat product | Included into analysis |  |
| MH429067 | GRA6 | 71_Gra6__W_III | Poland | NA | Raw meat product | Included into analysis |  |
| MH429066 | GRA6 | 35_Gra6__W_III | Poland | NA | Raw meat product | Included into analysis |  |
| MH429065 | GRA6 | 37_Gra6_W_III_ | Poland | NA | Raw meat product | Included into analysis |  |
| MH429064 | GRA6 | 84_Gra6_W__I_ | Poland | NA | Raw meat product | Included into analysis |  |
| MH429063 | GRA6 | 49_Gra6__A_I_II | Poland | NA | Raw meat product | Included into analysis |  |
| MH429062 | GRA6 | 22_Gra6_A_I_II | Poland | NA | Raw meat product | Included into analysis |  |
| MH429061 | GRA6 | 52_Gra6__W_II | Poland | NA | Raw meat product | Included into analysis |  |
| MH429060 | GRA6 | 51_Gra6__j_II | Poland | NA | Raw meat product | Included into analysis |  |
| MH429059 | GRA6 | 50_Gra6_j_II | Poland | NA | Raw meat product | Included into analysis |  |
| MH094810 | GRA6 | WB4 | Italy | Wild boar | Tissue | Included into analysis | [S86] |
| MG587987 | GRA6 | 335/11_fox_E9_E10_gra6 | Italy | Fox | Tissue | Included into analysis | [S29] |
| MG587986 | GRA6 | 324/11_fox_E3_E4_gra6 | Italy | Fox | Tissue | Included into analysis |  |
| MG587985 | GRA6 | 698/11_wild_boar_F7_F8_gra6 | Italy | Wild boar | Tissue | Included into analysis | [S29] |
| MG587984 | GRA6 | 847/11_wild_boar_F5_F6_gra6 | Italy | Wild boar | Tissue | Included into analysis |  |
| MG587983 | GRA6 | 846/11_wild_boar_F3_F4_gra6 | Italy | Wild boar | Tissue | Included into analysis |  |
| MG587982 | GRA6 | 699/11_wild_boar_F1_F2_gra6 | Italy | Wild boar | Tissue | Included into analysis |  |
| MG587981 | GRA6 | 12/11_roe_Deer_E11_E12_gra6 | Italy | Roe Deer | Tissue | Included into analysis |  |
| MG587980 | GRA6 | 339/11_fox_E7_E8_gra6 | Italy | Fox | Tissue | Included into analysis |  |
| MG587979 | GRA6 | 116/11_fox_E5_E6_gra6 | Italy | Fox | Tissue | Included into analysis |  |
| MG587978 | GRA6 | 806/11_fox_E1_E2_GRA6 | Italy | Fox | Tissue | Included into analysis |  |
| MG587977 | GRA6 | 807/11_wild_boar_H9_H10_gra6 | Italy | Wild boar | Tissue | Included into analysis |  |
| MG587976 | GRA6 | 723/11_wild_boar_H7_H8_gra6 | Italy | Wild boar | Tissue | Included into analysis |  |
| MG587975 | GRA6 | Q64_swine_H3_H4_gra6 | Italy | Pig | Tissue | Included into analysis |  |
| MG587974 | GRA6 | 730/11_wild_boar_G7_G8_gra6 | Italy | Wild boar | Tissue | Included into analysis |  |
| MG587973 | GRA6 | 700/11_wild_boar_G3_G4_gra6 | Italy | Wild boar | Tissue | Included into analysis |  |
| MG587972 | GRA6 | 6_bovine_F11_F12_gra6 | Italy | Cattle | Tissue | Included into analysis |  |
| MG587971 | GRA6 | Q68_swine_F9_F10_GRA6 | Italy | Pig | Tissue | Included into analysis |  |
| MG587970 | GRA6 | B8_swine_F7_F8_GRA6 | Italy | Pig | Tissue | Included into analysis |  |
| MG587969 | GRA6 | A7_swine_F5_F6_GRA6 | Italy | Pig | Tissue | Included into analysis |  |
| MG587968 | GRA6 | A4_swine_F3_F4_GRA6 | Italy | Pig | Tissue | Included into analysis |  |
| MG587967 | GRA6 | P54_swine_E11_E12_GRA6 | Italy | Pig | Tissue | Included into analysis |  |
| MG587966 | GRA6 | C5_swine_E9_E10_GRA6 | Italy | Pig | Tissue | Included into analysis |  |
| MG587965 | GRA6 | 783/11_wild_boar_D11_D12_GRA6 | Italy | Wild boar | Tissue | Included into analysis |  |
| MG587964 | GRA6 | 780/11_wild_boar_D9_D10_GRA6 | Italy | Wild boar | Tissue | Included into analysis |  |
| MG587963 | GRA6 | 869/11_fox_D7_D8_GRA6 | Italy | Fox | Tissue | Included into analysis |  |
| MG587962 | GRA6 | 876/11_fox_D3_D4_GRA6 | Italy | Fox | Tissue | Included into analysis | [S29] |
| MG587961 | GRA6 | 817/11_wild_boar_D1_D2_GRA6 | Italy | Wild boar | Tissue | Included into analysis |  |
| MG587960 | GRA6 | 91_bovine_B9_B10_GRA6 | Italy | Cattle | Tissue | Included into analysis |  |
| MG587959 | GRA6 | 64_bovine_B5_B6_GRA6 | Italy | Cattle | Tissue | Included into analysis |  |
| MG587958 | GRA6 | 41_bovine_B3_B4_GRA6 | Italy | Cattle | Tissue | Included into analysis |  |
| MG587957 | GRA6 | Q69_swine_G5_G6_gra6 | Italy | Pig | Tissue | Included into analysis |  |
| MG587956 | GRA6 | Q70_swine_G7_G8_gra6 | Italy | Pig | Tissue | Included into analysis |  |
| KY634426 | GRA6 | 53 | Poland | Goat | Milk | Included into analysis | [S26] |
| KY634425 | GRA6 | 45 | Poland | Goat | Milk | Included into analysis |  |
| KY634424 | GRA6 | 41 | Poland | Goat | Milk | Included into analysis |  |
| KY634423 | GRA6 | 36 | Poland | Goat | Milk | Included into analysis |  |
| KY634422 | GRA6 | 35 | Poland | Goat | Milk | Included into analysis |  |
| KY634421 | GRA6 | 30 | Poland | Goat | Milk | Included into analysis |  |
| KU599143 | GRA6 | TgPiPr09 | Portugal | Pig | Isolate | Included into analysis | [S122] |
| KU599142 | GRA6 | TgCkPr11 | Portugal | Chicken | Isolate | Included into analysis |  |
| KU599141 | GRA6 | TgPiPr13 | Portugal | Pig | Isolate | Included into analysis |  |
| KU599140 | GRA6 | TgPiPr05 | Portugal | Pig | Isolate | Included into analysis |  |
| KU599139 | GRA6 | TgPiPr07 | Portugal | Pig | Isolate | Included into analysis |  |
| KU599138 | GRA6 | TgCkPr03 | Portugal | Chicken | Isolate | Included into analysis |  |
| KU599137 | GRA6 | TgCkPr04 | Portugal | Chicken | Isolate | Included into analysis |  |
| KU599136 | GRA6 | TgPiPr14 | Portugal | Pig | Isolate | Included into analysis |  |
| KU599135 | GRA6 | TgCkPr01 | Portugal | Chicken | Isolate | Included into analysis |  |
| KU599134 | GRA6 | TgCkPr02 | Portugal | Chicken | Isolate | Included into analysis |  |
| KU599133 | GRA6 | TgCkPr16 | Portugal | Chicken | Isolate | Included into analysis | [S122] |
| KU599132 | GRA6 | TgPiPr02 | Portugal | Pig | Isolate | Included into analysis |  |
| KU599076 | GRA6 | FR-OVI-ARI022-(TgA32129) | France | Sheep | Isolate | Included into analysis |  |
| KU599075 | GRA6 | FR-OVI-ARI043-(TgA32109) | France | Sheep | Isolate | Included into analysis |  |
| KU599074 | GRA6 | FR-OVI-ARI025-(TgA32091) | France | Sheep | Isolate | Included into analysis |  |
| KU599073 | GRA6 | FR-OVI-ARI033-(TgA32099) | France | Sheep | Isolate | Included into analysis |  |
| KU599072 | GRA6 | FR-OVI-ARI027-(TgA32093) | France | Sheep | Isolate | Included into analysis |  |
| KU599071 | GRA6 | FR-OVI-ARI029-(TgA32095) | France | Sheep | Isolate | Included into analysis |  |
| KU599070 | GRA6 | FR-OVI-ARI042-(TgA32108) | France | Sheep | Isolate | Included into analysis |  |
| KU599069 | GRA6 | FR-OVI-ARI050-(TgA32116) | France | Sheep | Isolate | Included into analysis |  |
| KU599068 | GRA6 | FR-OVI-ARI021-(TgA32088) | France | Sheep | Isolate | Included into analysis |  |
| KU599067 | GRA6 | FR-OVI-ARI026-(TgA32092) | France | Sheep | Isolate | Included into analysis |  |
| KU599066 | GRA6 | FR-OVI-ARI049-(TgA32115) | France | Sheep | Isolate | Included into analysis |  |
| KU599065 | GRA6 | FR-OVI-ARI056-(TgA32122) | France | Sheep | Isolate | Included into analysis |  |
| GU325791 | GRA6 | H78-13 | The Netherlands | Sheep | Tissue | Included into analysis | [S49] |
| GU325790 | GRA6 | H78-07 | The Netherlands | Sheep | Tissue | Included into analysis |  |
| KN044667 | GRA6 | MAS | France | Human | Isolate | Excluded, European isolate likely related to migration/importation | NA |
| AEXC02002701 | GRA6 | MAS | France | Human | Isolate | Excluded, European isolate likely related to migration/importation | NA |
| KN000290 | GRA6 | FOU | France | Human | Isolate | Excluded, European isolate likely related to migration/importation | NA |
| AEYH02002992 | GRA6 | FOU | France | Human | Isolate | Excluded, European isolate likely related to migration/importation | NA |
| DQ459452 | GRA7 | RMS-1994-LEF | France | Human | Isolate | Included into analysis | [S137] |
| EF639859 | GRA7 | RMS-2003-DJO | France | Human | Isolate | Included into analysis | [S137] |
| MT361129 | GRA7 | TgShSp24 | Spain | Sheep | Tissue | Included into analysis | [S48] |
| MT361128 | GRA7 | ID_18-15_21 | Spain | Sheep | Fetal tissue | Included into analysis |  |
| MT361127 | GRA7 | ID_18-15_20 | Spain | Sheep | Fetal tissue | Included into analysis |  |
| KU599311 | GRA7 | TgPiPr09 | Portugal | Pig | Isolate | Included into analysis | [S122] |
| KU599310 | GRA7 | TgCkPr11 | Portugal | chicken | Isolate | Included into analysis |  |
| KU599309 | GRA7 | TgPiPr13 | Portugal | Pig | Isolate | Included into analysis |  |
| KU599308 | GRA7 | TgPiPr05 | Portugal | Pig | Isolate | Included into analysis |  |
| KU599307 | GRA7 | TgPiPr07 | Portugal | Pig | Isolate | Included into analysis |  |
| KU599306 | GRA7 | TgCkPr03 | Portugal | chicken | Isolate | Included into analysis |  |
| KU599305 | GRA7 | TgCkPr04 | Portugal | chicken | Isolate | Included into analysis |  |
| KU599304 | GRA7 | TgPiPr14 | Portugal | Pig | Isolate | Included into analysis |  |
| KU599303 | GRA7 | TgCkPr01 | Portugal | chicken | Isolate | Included into analysis |  |
| KU599302 | GRA7 | TgCkPr02 | Portugal | chicken | Isolate | Included into analysis |  |
| KU599301 | GRA7 | TgCkPr16 | Portugal | chicken | Isolate | Included into analysis |  |
| KU599300 | GRA7 | TgPiPr02 | Portugal | Pig | Isolate | Included into analysis |  |
| KU599244 | GRA7 | FR-OVI-ARI022-(TgA32129) | France | Sheep | Isolate | Included into analysis |  |
| KU599243 | GRA7 | FR-OVI-ARI043-(TgA32109) | France | Sheep | Isolate | Included into analysis |  |
| KU599242 | GRA7 | FR-OVI-ARI025-(TgA32091) | France | Sheep | Isolate | Included into analysis |  |
| KU599241 | GRA7 | FR-OVI-ARI033-(TgA32099) | France | Sheep | Isolate | Included into analysis |  |
| KU599240 | GRA7 | FR-OVI-ARI027-(TgA32093) | France | Sheep | Isolate | Included into analysis |  |
| KU599239 | GRA7 | FR-OVI-ARI029-(TgA32095) | France | Sheep | Isolate | Included into analysis |  |
| KU599238 | GRA7 | FR-OVI-ARI042-(TgA32108) | France | Sheep | Isolate | Included into analysis |  |
| KU599237 | GRA7 | FR-OVI-ARI050-(TgA32116) | France | Sheep | Isolate | Included into analysis | [S122] |
| KU599236 | GRA7 | FR-OVI-ARI021-(TgA32088) | France | Sheep | Isolate | Included into analysis |  |
| KU599235 | GRA7 | FR-OVI-ARI026-(TgA32092) | France | Sheep | Isolate | Included into analysis |  |
| KU599234 | GRA7 | FR-OVI-ARI049-(TgA32115) | France | Sheep | Isolate | Included into analysis |  |
| KU599233 | GRA7 | FR-OVI-ARI056-(TgA32122) | France | Sheep | Isolate | Included into analysis |  |
| KN044635 | GRA7 | MAS | France | Human | Isolate | Excluded, European isolate likely related to migration/importation | NA |
| AEXC02001945 | GRA7 | MAS | France | Human | Isolate | Excluded, European isolate likely related to migration/importation | NA |
| KN003101 | GRA7 | FOU | France | Human | Isolate | Excluded, European isolate likely related to migration/importation | NA |
| AEYH02000783 | GRA7 | FOU | France | Human | Isolate | Excluded, European isolate likely related to migration/importation | NA |
| DQ459452 | GRA7 | RMS-1994-LEF | France | Human | Isolate | Included into analysis | [S137] |
| EF639859 | GRA7 | RMS-2003-DJO | France | Human | Isolate | Included into analysis |  |
| EF639858 | GRA7 | FR1-2002-Cap cap 01 | France | Deer | Isolate | Included into analysis |  |
| EF639857 | GRA7 | FR1-2002-Sus scr 02 | France | Wild boar | Isolate | Included into analysis |  |
| EF626970 | GRA7 | RMS-2001-WAU | France | NA | Isolate | Included into analysis |  |
| EF626969 | GRA7 | RMS-2001-GRA | France | NA | Isolate | Included into analysis |  |
| EF626968 | GRA7 | RMS-2000-GIL | France | NA | Isolate | Included into analysis |  |
| EF626967 | GRA7 | RMS-2000-BAR | France | NA | Isolate | Included into analysis |  |
| EF626966 | GRA7 | RMS-1995-ABE | France | NA | Isolate | Included into analysis |  |
| EF626965 | GRA7 | RMS-1999-GUI | France | NA | Isolate | Included into analysis |  |
| DQ465955 | GRA7 | RMS-2001-MAU | France | NA | Isolate | Included into analysis |  |
| DQ459454 | GRA7 | RMS-1997-PAR | France | NA | Isolate | Included into analysis |  |
| DQ459453 | GRA7 | MAR-2000-HOU | France | NA | Isolate | Included into analysis | [S137] |
| DQ459449 | GRA7 | GRE-2000-BRA | France | NA | Isolate | Included into analysis |  |
| DQ459448 | GRA7 | TOU-2004-MON | France | NA | Isolate | Included into analysis |  |
| DQ459447 | GRA7 | RMS-1999-BES | France | NA | Isolate | Included into analysis |  |
| DQ459446 | GRA7 | RMS-2000-TRA | France | NA | Isolate | Included into analysis |  |
| DQ459452 | GRA7 | RMS-1994-LEF | France | NA | Isolate | Included into analysis |  |
| EF639859 | GRA7 | RMS-2003-DJO | France | NA | Isolate | Included into analysis |  |
| MN275931 | ROP8 | PRU-ROP8-Limoges | France | Human | Isolate | Included into analysis | [S138] |
| MN275919 | B1 | PRU-B-Limoges | France | PRU_Type_II | Isolate | Included into analysis |  |
| MT019620 | B1 | ID 60322 | Italy | Otter | Tissue | Included into analysis | [S135] |
| MH094814 | B1 | WB54 | Italy | Wild boar | Tissue | Excluded, likely sequencing errors in terminal parts of the sequence | [S86] |
| MH094813 | B1 | WB25 | Italy | Wild boar | Tissue | Excluded, likely sequencing errors in terminal parts of the sequence |  |
| MH094812 | B1 | WB4 | Italy | Wild boar | Tissue | Excluded, likely sequencing errors in terminal parts of the sequence |  |
| MG574970 | B1 | Pigs | Italy | Pig | Tissue | Excluded, likely sequencing errors in terminal parts of the sequence | [S36] |
| MG574969 | B1 | Pigs | Italy | Pig | Tissue | Excluded, likely sequencing errors in terminal parts of the sequence |  |
| MG574968 | B1 | Pigs | Italy | Pig | Tissue | Excluded, likely sequencing errors in terminal parts of the sequence |  |
| MG574967 | B1 | Pigs | Italy | Pig | Tissue | Excluded, likely sequencing errors in terminal parts of the sequence |  |
| MG574966 | B1 | Pigs | Italy | Pig | Tissue | Excluded, likely sequencing errors in terminal parts of the sequence |  |
| MG574965 | B1 | Pigs | Italy | Pig | Tissue | Excluded, likely sequencing errors in terminal parts of the sequence |  |
| MG574964 | B1 | Pigs | Italy | Pig | Tissue | Excluded, likely sequencing errors in terminal parts of the sequence | [S36] |
| MG574963 | B1 | Pigs | Italy | Pig | Tissue | Excluded, likely sequencing errors in terminal parts of the sequence |  |
| MG574962 | B1 | Pigs | Italy | Pig | Tissue | Excluded, likely sequencing errors in terminal parts of the sequence |  |
| KY706491 | B1 | Italy | Italy | Molluscs | Tissue | Excluded, likely sequencing errors in terminal parts of the sequence | [S139] |
| MF576258 | B1 | OWM22-2017 | Poland | Sheep | Milk | Included into analysis | NA |
| MF576257 | B1 | OWM8-2017 | Poland | Sheep | Milk | Included into analysis | NA |
| MF576256 | B1 | OWM4-2017 | Poland | Sheep | Milk | Included into analysis | NA |
| MF576255 | B1 | AF24-2017 | Poland | Yellow-necked mouse | Tissue | Included into analysis | NA |
| MF576254 | B1 | DZK18-2017 | Poland | Wild boar | Tissue | Included into analysis | NA |
| MF576253 | B1 | SAR14_2016 | Poland | Roe deer | Tissue | Included into analysis | NA |
| MF576252 | B1 | SAR12-2016 | Poland | Roe deer | Tissue | Included into analysis | NA |
| MF576251 | B1 | SAR10-2016 | Poland | Roe deer | Tissue | Included into analysis | NA |
| MF576250 | B1 | SAR8-2016 | Poland | Roe deer | Tissue | Included into analysis | NA |
| MF576249 | B1 | SAR7-2016 | Poland | Roe deer | Tissue | Included into analysis | NA |
| MF576248 | B1 | SAR2-2016 | Poland | Roe deer | Tissue | Included into analysis | NA |
| MF576247 | B1 | SAR1-2016 | Poland | Roe deer | Tissue | Included into analysis | NA |
| MF576246 | B1 | CHD12-2016 | Poland | Wild boar | Tissue | Included into analysis | NA |
| MF576245 | B1 | CHD10-2016 | Poland | Wild boar | Tissue | Included into analysis | NA |
| MF576244 | B1 | BA7-2016 | Poland | Sheep | Tissue | Included into analysis | NA |
| MF576243 | B1 | BA6-2016 | Poland | Sheep | Tissue | Included into analysis | NA |
| MF576242 | B1 | BA4-2016 | Poland | Sheep | Tissue | Included into analysis | NA |
| MF576241 | B1 | BA1-2016 | Poland | Sheep | Tissue | Included into analysis | NA |
| MF576240 | B1 | CAT1-2016 | Poland | Cat | Faeces | Included into analysis | NA |
| MF576239 | B1 | 2971-2017 | Poland | Human | Amniotic fluid | Included into analysis | NA |
| MF576238 | B1 | 8307-2016 | Poland | Human | Amniotic fluid | Included into analysis | NA |
| MF576237 | B1 | 6506-2016 | Poland | Human | Amniotic fluid | Included into analysis | NA |
| MF576236 | B1 | 6716-2016 | Poland | Human | Amniotic fluid | Included into analysis | NA |
| MF576235 | B1 | 6382-2016 | Poland | Human | Amniotic fluid | Included into analysis | NA |
| MF576234 | B1 | 2706.1-2016 | Poland | Human | Amniotic fluid | Included into analysis | NA |
| MF576233 | B1 | 3748-2015 | Poland | Human | Amniotic fluid | Included into analysis | NA |
| MF576232 | B1 | 1224-2015 | Poland | Human | Amniotic fluid | Included into analysis | NA |
| MF576231 | B1 | 3874-2014 | Poland | Human | Amniotic fluid | Included into analysis | NA |
| MF576230 | B1 | 0849-2014 | Poland | Human | Amniotic fluid | Included into analysis | NA |
| MF576229 | B1 | 4833-2013 | Poland | Human | Amniotic fluid | Included into analysis | NA |
| MF576228 | B1 | 4724-2013 | Poland | Human | Amniotic fluid | Included into analysis | NA |
| MF576227 | B1 | 2481-2013 | Poland | Human | Amniotic fluid | Included into analysis | NA |
| MF576226 | B1 | 7593-2012 | Poland | Human | Amniotic fluid | Included into analysis | NA |
| MF576225 | B1 | 3688-2012 | Poland | Human | Amniotic fluid | Included into analysis | NA |
| MF576224 | B1 | 2532-2012 | Poland | Human | Amniotic fluid | Included into analysis | NA |
| MF576223 | B1 | 373-2012 | Poland | Human | Amniotic fluid | Included into analysis | NA |
| MF576222 | B1 | O(26)-2011 | Poland | Human | Amniotic fluid | Included into analysis | NA |
| MF576221 | B1 | I(9129)-2009 | Poland | Human | Amniotic fluid | Included into analysis | NA |
| MF576220 | B1 | D(7054)-2008 | Poland | Human | Amniotic fluid | Included into analysis | NA |
| MF576219 | B1 | R-2007 | Poland | Human | Amniotic fluid | Included into analysis | NA |
| MF576218 | B1 | G-2007 | Poland | Human | Amniotic fluid | Included into analysis | NA |
| MF576217 | B1 | B(1755)-2007 | Poland | Human | Amniotic fluid | Included into analysis | NA |
| MF576216 | B1 | P-2006 | Poland | Human | Amniotic fluid | Included into analysis | NA |
| MF576215 | B1 | N(97)-2006 | Poland | Human | Amniotic fluid | Included into analysis | NA |
| MF576214 | B1 | E(77)-2006 | Poland | Human | Amniotic fluid | Included into analysis | NA |
| MF543572 | B1 | F-2006 | Poland | Human | Amniotic fluid | Included into analysis | NA |
| KX944482 | B1 | 836-L-IMNO | Poland | Ixodid tick | Tick | Included into analysis | [S99] |
| KX944481 | B1 | 782-L-IMNO | Poland | Ixodid tick | Tick | Included into analysis |  |
| KX944480 | B1 | 781-L-IMNO | Poland | Ixodid tick | Tick | Included into analysis |  |
| KX944479 | B1 | 774-L-IMNO | Poland | Ixodid tick | Tick | Included into analysis |  |
| KY554828 | B1 | KL | Italy | NA | Ready to eat salad | Included into analysis | [S93] |
| KY554827 | B1 | NJ | Italy | NA | Ready to eat salad | Included into analysis |  |
| KY554826 | B1 | HG | Italy | NA | Ready to eat salad | Included into analysis |  |
| KY554825 | B1 | BG | Italy | NA | Ready to eat salad | Included into analysis |  |
| KY554824 | B1 | MM | Italy | NA | Ready to eat salad | Included into analysis |  |
| KU748893 | B1 | TG-KLR-720-IMNO | Poland | Questing Ixodid tick | Tick | Included into analysis | [S99] |
| KU748892 | B1 | TG-KLR-631-IMNO | Poland | Questing Ixodid tick | Tick | Included into analysis |  |
| KU748891 | B1 | TG-KLR-625-IMNO | Poland | Questing Ixodid tick | Tick | Included into analysis |  |
| KU748890 | B1 | TG-KLR-610-IMNO | Poland | Questing Ixodid tick | Tick | Included into analysis |  |
| KU748889 | B1 | TG-KLR-583-IMNO | Poland | Questing Ixodid tick | Tick | Included into analysis |  |
| KU748888 | B1 | TG-KLR-555-IMNO | Poland | Questing Ixodid tick | Tick | Included into analysis |  |
| KU748887 | B1 | TG-KLK-1018-IMNO | Poland | Fed Ixodid tick | Tick | Included into analysis |  |
| KU748886 | B1 | TG-KLK-983-IMNO | Poland | Fed Ixodid tick | Tick | Included into analysis |  |
| KU748885 | B1 | TG-KLK-905-IMNO | Poland | Fed Ixodid tick | Tick | Included into analysis | [S99] |
| KU748884 | B1 | TG-KLK-897-IMNO | Poland | Fed Ixodid tick | Tick | Included into analysis |  |
| KU748883 | B1 | TG-KLK-830-IMNO | Poland | Fed Ixodid tick | Tick | Included into analysis |  |
| KU748882 | B1 | TG-KLK-365-IMNO | Poland | Fed Ixodid tick | Tick | Included into analysis |  |
| KT266796 | B1 | TG42-ZEL-MDW | Poland | NA | Water | Included into analysis | [S95] |
| KT266795 | B1 | TG41-SZN-PLO | Poland | NA | Water | Included into analysis |  |
| KT266794 | B1 | TG40-SZN-JST | Poland | NA | Water | Included into analysis |  |
| KT266793 | B1 | TG38-SZN-SBR | Poland | NA | Water | Included into analysis |  |
| KT266792 | B1 | TG34-SWU-SWN | Poland | NA | Water | Included into analysis |  |
| KT266791 | B1 | TG18-TRZ-JM | Poland | NA | Water | Included into analysis |  |
| KT266790 | B1 | TG1-SZN-GL | Poland | NA | Water | Included into analysis |  |
| KR559682 | B1 | TGPC | Slovakia | Cormorant | Tissue | Excluded, likely sequencing errors in parts of the sequence | [S140] |
| KN044615 | B1 | MAS | France | Human | Isolate | Excluded, European isolate likely related to migration/importation | NA |
| KN044618 | B1 | MAS | France | Human | Isolate | Excluded, European isolate likely related to migration/importation | NA |
| AEXC02000676 | B1 | MAS | France | Human | Isolate | Excluded, European isolate likely related to migration/importation | NA |
| AEXC02000959 | B1 | MAS | France | Human | Isolate | Excluded, European isolate likely related to migration/importation | NA |
| AEXC02000960 | B1 | MAS | France | Human | Isolate | Excluded, European isolate likely related to migration/importation | NA |
| AEXC02000961 | B1 | MAS | France | Human | Isolate | Excluded, European isolate likely related to migration/importation | NA |
| AEXC02000962 | B1 | MAS | France | Human | Isolate | Excluded, European isolate likely related to migration/importation | NA |
| AEXC02000963 | B1 | MAS | France | Human | Isolate | Excluded, European isolate likely related to migration/importation | NA |
| KN000238 | B1 | FOU | France | Human | Isolate | Excluded, European isolate likely related to migration/importation | NA |
| KN000255 | B1 | FOU | France | Human | Isolate | Excluded, European isolate likely related to migration/importation | NA |
| AEYH02000709 | B1 | FOU | France | Human | Isolate | Excluded, European isolate likely related to migration/importation | NA |
| AEYH02002329 | B1 | FOU | France | Human | Isolate | Excluded, European isolate likely related to migration/importation | NA |
| AEYH02002330 | B1 | FOU | France | Human | Isolate | Excluded, European isolate likely related to migration/importation | NA |
| AEYH02002331 | B1 | FOU | France | Human | Isolate | Excluded, European isolate likely related to migration/importation | NA |
| AEYH02002332 | B1 | FOU | France | Human | Isolate | Excluded, European isolate likely related to migration/importation | NA |
| MH606170 | BTUB | 53_BTUB_A_I/III | Poland | NA | Raw meat product | Included into analysis | [S136] |
| MH606169 | BTUB | 24_BTUB_A_I/III | Poland | NA | Raw meat product | Included into analysis |  |
| MH094811 | BTUB | WB4 | Italy | Wild boar | Tissue | Included into analysis | [S86] |
| MG588012 | BTUB | 817/11_wild_boar_A1_A2_BTUB | Italy | Wild boar | Tissue | Included into analysis | [S29] |
| KY634428 | BTUB | 21 | Poland | Goat | Milk | Included into analysis | [S26] |
| KY634427 | BTUB | 20 | Poland | Goat | Milk | Included into analysis |  |
| MN958072 | SAG1 | ID60322 | Italy | Otter | Tissue | Included into analysis | [S135] |
| MH606165 | SAG1 | 97_SAG1_W_III | Poland | Raw meat product | Raw meat product | Included into analysis | [S136] |
| MH606164 | SAG1 | 93_SAG1_A_I | Poland | Raw meat product | Raw meat product | Included into analysis |  |
| MH606163 | SAG1 | 54_SAG1_W_I | Poland | Raw meat product | Raw meat product | Included into analysis |  |
| MH606162 | SAG1 | 41_SAG1_W_II/III | Poland | Raw meat product | Raw meat product | Included into analysis |  |
| MH606161 | SAG1 | 27_SAG1_W_III | Poland | Raw meat product | Raw meat product | Included into analysis | [S136] |
| MH606160 | SAG1 | 26_SAG1_A_I/III | Poland | Raw meat product | Raw meat product | Included into analysis |  |
| MH606159 | SAG1 | 25_SAG1_A_II/III | Poland | Raw meat product | Raw meat product | Included into analysis |  |
| MH606158 | SAG1 | 19_SAG1_A_II/III | Poland | Raw meat product | Raw meat product | Included into analysis |  |
| MH606157 | SAG1 | 18_SAG1_A_III | Poland | Raw meat product | Raw meat product | Included into analysis |  |
| MH606156 | SAG1 | 17_SAG1_A_II/III | Poland | Raw meat product | Raw meat product | Included into analysis |  |
| MH606155 | SAG1 | 11_SAG1_A_I | Poland | Raw meat product | Raw meat product | Included into analysis |  |
| MH606154 | SAG1 | 9_SAG1_A_I | Poland | Raw meat product | Raw meat product | Included into analysis |  |
| MH606153 | SAG1 | 6_SAG1_A_II/III | Poland | Raw meat product | Raw meat product | Included into analysis |  |
| MH606152 | SAG1 | 3_SAG1_A_II/III | Poland | Raw meat product | Raw meat product | Included into analysis |  |
| MH606151 | SAG1 | 1_SAG1_A_I | Poland | Raw meat product | Raw meat product | Included into analysis |  |
| MG588014 | SAG1 | 876/11_fox_F1_F2_SAG1 | Italy | Fox | Tissue | Included into analysis | [S29] |
| MG588013 | SAG1 | 817/11_wild_boar_E7_E8_SAG1 | Italy | Wild boar | Tissue | Included into analysis |  |
| KY634430 | SAG1 | 17 | Poland | Goat | Milk | Included into analysis | [S26] |
| KY634429 | SAG1 | 43 | Poland | Goat | Milk | Included into analysis |  |
| GQ253098 | SAG1 | RMS-2000-ROU | France | Human | Isolate | Included into analysis | NA |
| GQ253097 | SAG1 | RMS-1998-BOU | France | Human | Isolate | Included into analysis | NA |
| GQ253096 | SAG1 | RMS-1999-GUI | France | Human | Isolate | Included into analysis | NA |
| GQ253095 | SAG1 | RMS-1999-BOR | France | Human | Isolate | Included into analysis | NA |
| GQ253094 | SAG1 | RMS-1999-BOUC | France | Human | Isolate | Included into analysis | NA |
| GQ253093 | SAG1 | RMS-2000-PER | France | Human | Isolate | Included into analysis | NA |
| GQ253092 | SAG1 | RMS-2000-GIL | France | Human | Isolate | Included into analysis | NA |
| GQ253091 | SAG1 | RMS-1998-ROB | France | Human | Isolate | Included into analysis | NA |
| GQ253090 | SAG1 | RMS-2000-CON | France | Human | Isolate | Included into analysis | NA |
| GQ253089 | SAG1 | RMS-1994-COE | France | Human | Isolate | Included into analysis | NA |
| GQ253088 | SAG1 | RMS-1999-RUN | France | Human | Isolate | Included into analysis | NA |
| GQ253087 | SAG1 | RMS-2000-DAF | France | Human | Isolate | Included into analysis | NA |
| MN958064 | altSAG2 | ID60322 | Italy | Otter | Tissue | Included into analysis | [S135] |
| MN958063 | 5'SAG2 | ID60322 | Italy | Otter | Tissue | Included into analysis |  |
| MN958062 | 3'SAG2 | ID60322 | Italy | Otter | Tissue | Included into analysis |  |
| MH606168 | altSAG2 | 77_altSAG2_W_II | Poland | Raw meat product |  | Included into analysis | [S136] |
| MH606167 | altSAG2 | 66_altSAG2_W_III | Poland | Raw meat product |  | Included into analysis |  |
| MH606166 | altSAG2 | 58_altSAG2_W_III | Poland | Raw meat product |  | Included into analysis |  |
| MH606150 | 3'SAG2 | 55_3_SAG2_W_III | Poland | Raw meat product |  | Included into analysis |  |
| MH606149 | 3'SAG2 | 7_3_SAG2_A_III | Poland | Raw meat product |  | Included into analysis |  |
| AY707931 | SAG2 | 16 | Portugal | Human | Isolate | Included into analysis | [S13] |
| AY707930 | SAG2 | 14 | Portugal | Human | Isolate | Included into analysis |  |
| AY707929 | SAG2 | 12 | Portugal | Human | Isolate | Included into analysis |  |
| AY707928 | SAG2 | 10 | Portugal | Human | Isolate | Included into analysis |  |
| AY707927 | SAG2 | 6 | Portugal | Human | Isolate | Included into analysis |  |
| AY707926 | SAG2 | 4 | Portugal | Human | Isolate | Included into analysis |  |
| AY707925 | SAG2 | 3 | Portugal | Human | Isolate | Included into analysis |  |
| MN958073 | SAG3 | ID60322 | Italy | Otter | Tissue | Included into analysis | [S135] |
| MT361130 | SAG3 | TgShSp24 | Spain | Sheep | Tissue | Included into analysis | [S48] |
| MT361126 | SAG3 | ID_15-121_1 | Spain | Sheep | Fetal tissue | Included into analysis |  |
| MT361125 | SAG3 | ID_17-4_2 | Spain | Sheep | Fetal tissue | Included into analysis |  |
| MT361124 | SAG3 | ID_17-28_1 | Spain | Sheep | Fetal tissue | Included into analysis | [S48] |
| MT358429 | SAG3 | ID#17/21.1 | Spain | Sheep | Fetal tissue | Included into analysis |  |
| KU599479 | SAG3 | TgPiPr09 | Portugal | Pig | Isolate | Included into analysis | [S122] |
| KU599478 | SAG3 | TgCkPr11 | Portugal | Chicken | Isolate | Included into analysis |  |
| KU599477 | SAG3 | TgPiPr13 | Portugal | Pig | Isolate | Included into analysis |  |
| KU599476 | SAG3 | TgPiPr05 | Portugal | Pig | Isolate | Included into analysis |  |
| KU599475 | SAG3 | TgPiPr07 | Portugal | Pig | Isolate | Included into analysis |  |
| KU599474 | SAG3 | TgCkPr03 | Portugal | Chicken | Isolate | Included into analysis |  |
| KU599473 | SAG3 | TgCkPr04 | Portugal | Chicken | Isolate | Included into analysis |  |
| KU599472 | SAG3 | TgPiPr14 | Portugal | Pig | Isolate | Included into analysis |  |
| KU599471 | SAG3 | TgCkPr01 | Portugal | Chicken | Isolate | Included into analysis |  |
| KU599470 | SAG3 | TgCkPr02 | Portugal | Chicken | Isolate | Included into analysis |  |
| KU599469 | SAG3 | TgCkPr16 | Portugal | Chicken | Isolate | Included into analysis |  |
| KU599468 | SAG3 | TgPiPr02 | Portugal | Pig | Isolate | Included into analysis |  |
| KU599412 | SAG3 | FR-OVI-ARI022-(TgA32129) | France | Sheep | Isolate | Included into analysis |  |
| KU599411 | SAG3 | FR-OVI-ARI043-(TgA32109) | France | Sheep | Isolate | Included into analysis |  |
| KU599410 | SAG3 | FR-OVI-ARI025-(TgA32091) | France | Sheep | Isolate | Included into analysis |  |
| KU599409 | SAG3 | FR-OVI-ARI033-(TgA32099) | France | Sheep | Isolate | Included into analysis |  |
| KU599408 | SAG3 | FR-OVI-ARI027-(TgA32093) | France | Sheep | Isolate | Included into analysis |  |
| KU599407 | SAG3 | FR-OVI-ARI029-(TgA32095) | France | Sheep | Isolate | Included into analysis |  |
| KU599406 | SAG3 | FR-OVI-ARI042-(TgA32108) | France | Sheep | Isolate | Included into analysis |  |
| KU599405 | SAG3 | FR-OVI-ARI050-(TgA32116) | France | Sheep | Isolate | Included into analysis |  |
| KU599404 | SAG3 | FR-OVI-ARI021-(TgA32088) | France | Sheep | Isolate | Included into analysis |  |
| KU599403 | SAG3 | FR-OVI-ARI026-(TgA32092) | France | Sheep | Isolate | Included into analysis | [S122] |
| KU599402 | SAG3 | FR-OVI-ARI049-(TgA32115) | France | Sheep | Isolate | Included into analysis |  |
| KU599401 | SAG3 | FR-OVI-ARI056-(TgA32122) | France | Sheep | Isolate | Included into analysis |  |
| MW132601 | cs3 | TgPigSp4 | Spain | Pig | Tissue | Included into analysis | [S42] |
| MW132600 | cs3 | TgPigSp1 | Spain | Pig | Tissue | Included into analysis |  |
| MN958071 | PK1 | ID60322 | Italy | Otter | Tissue | Included into analysis | [S135] |
| MN958067 | c22-8 | ID60322 | Italy | Otter | Tissue | Included into analysis |  |
| MG588017 | c22-8 | 876/11_fox_G1_G2_C22-8 | Italy | Fox | Tissue | Included into analysis | [S29] |
| MG588016 | c22-8 | 817/11_wild_boar_E3_E4_C22-8 | Italy | Wild boar | Tissue | Included into analysis |  |
| MG588015 | c22-8 | 335/11_fox_C7_C8_C22-8 | Italy | Fox | Tissue | Included into analysis |  |
| MH606148 | 3'SAG2 | 4_3SAG2_A_III | Poland | Raw meat product | Raw meat product | Included into analysis | [S136] |
| MH606171 | SAG3 | NA | Poland | Raw meat product | Raw meat product | Included into analysis |  |
| MH606172 | SAG3 | NA | Poland | Raw meat product | Raw meat product | Included into analysis |  |
| MH606173 | SAG3 | NA | Poland | Raw meat product | Raw meat product | Included into analysis |  |
| MH606174 | SAG3 | NA | Poland | Raw meat product | Raw meat product | Included into analysis |  |
| MH606175 | SAG3 | NA | Poland | Raw meat product | Raw meat product | Included into analysis |  |
| MH606176 | SAG3 | NA | Poland | Raw meat product | Raw meat product | Included into analysis |  |
| MH606177 | SAG3 | NA | Poland | Raw meat product | Raw meat product | Included into analysis |  |
| MH606178 | SAG3 | NA | Poland | Raw meat product | Raw meat product | Included into analysis |  |
| MH606179 | SAG3 | NA | Poland | Raw meat product | Raw meat product | Included into analysis |  |
| MH606180 | SAG3 | NA | Poland | Raw meat product | Raw meat product | Included into analysis |  |
| MH606181 | SAG3 | NA | Poland | Raw meat product | Raw meat product | Included into analysis |  |
| MH606182 | SAG3 | NA | Poland | Raw meat product | Raw meat product | Included into analysis |  |
| MH606183 | SAG3 | NA | Poland | Raw meat product | Raw meat product | Included into analysis | [S136] |
| MH606184 | SAG3 | NA | Poland | Raw meat product | Raw meat product | Included into analysis |  |
| MG588011 | altSAG2 | 41_bovino_G3_G4_altSAG2 | Italy | Cattle | Tissue | Included into analysis | [S29] |
| MG588010 | altSAG2 | 697/11_wild_boar_D9_D10_altSAG2 | Italy | Wild boar | Tissue | Included into analysis |  |
| MG588009 | altSAG2 | 698/11_wild_boar_D11_D12_altSAG2 | Italy | Wild boar | Tissue | Included into analysis |  |
| MG588008 | altSAG2 | 847/11_wild_boar_D7_D8_altSAG2 | Italy | Wild boar | Tissue | Included into analysis |  |
| MG588007 | altSAG2 | 846/11_wild_boar_D5_D6_altSAG2 | Italy | Wild boar | Tissue | Included into analysis |  |
| MG588006 | altSAG2 | 699/11_wild_boar_D3_D4_altSAG2 | Italy | Wild boar | Tissue | Included into analysis |  |
| MG588005 | altSAG2 | 12/11_roe_Deer_D1_D2_altSAG2 | Italy | Roe Deer | Tissue | Included into analysis |  |
| MG588004 | altSAG2 | 339/11_fox_C11_C12_altSAG2 | Italy | Fox | Tissue | Included into analysis |  |
| MG588003 | altSAG2 | 116/11_fox_C9_C10_altSAG2 | Italy | Fox | Tissue | Included into analysis |  |
| MG588002 | altSAG2 | 806/11_fox_C7_C8_altSAG2 | Italy | Fox | Tissue | Included into analysis |  |
| MG588001 | altSAG2 | 63_bovine_H5_H6_altSAG2 | Italy | Cattle | Tissue | Included into analysis |  |
| MG588000 | altSAG2 | Q64_swine_H1_H2_altSAG2 | Italy | Pig | Tissue | Included into analysis |  |
| MG587999 | altSAG2 | 766/11_wild_boar_G9_G10_altSAG2 | Italy | Wild boar | Tissue | Included into analysis |  |
| MG587998 | altSAG2 | 730/11_wild_boar_G5_G6_altSAG2 | Italy | Wild boar | Tissue | Included into analysis |  |
| MG587997 | altSAG2 | 700/11_wild_boar_G1_G2_altSAG2 | Italy | Wild boar | Tissue | Included into analysis |  |
| MG587996 | altSAG2 | 6_bovine_F9_F10_altSAG2 | Italy | Cattle | Tissue | Included into analysis |  |
| MG587995 | altSAG2 | 41_bovine_G3_G4_altSAG2 | Italy | Cattle | Tissue | Included into analysis |  |
| MG587994 | 5'SAG2 | 876/11_fox_F11_F12_5'SAG2 | Italy | Fox | Tissue | Included into analysis |  |
| MG587993 | 5'SAG2 | 817/11_wild_boar_E5_E6_5'SAG2 | Italy | Wild boar | Tissue | Included into analysis |  |
| MG587992 | altSAG2 | 780/11_wild_boar_E1_E2_altSAG2 | Italy | Wild boar | Tissue | Included into analysis |  |
| MG587991 | altSAG2 | 876/11_fox_D5_D6_altSAG2 | Italy | Fox | Tissue | Included into analysis |  |
| MG587990 | altSAG2 | 817/11_wild_boar_C11_C12_altSAG2 | Italy | Wild boar | Tissue | Included into analysis | [S29] |
| MG587989 | 5'SAG2 | 63_bovine_H3_H4_5'SAG2 | Italy | Cattle | Tissue | Included into analysis |  |
| MG587988 | altSAG2 | 890/11_wild_boar_G3_G4_altSAG2 | Italy | Wild boar | Tissue | Included into analysis |  |
| MN958066 | B-TUB | ID60322 | Italy | Otter | Tissue | Included into analysis | [S135] |
| MN958065 | Apico | ID60322 | Italy | Otter | Tissue | Included into analysis |  |
| MN958067 | C22-8 | ID60322 | Italy | Otter | Tissue | Included into analysis |  |
| MN958068 | C29 | ID60322 | Italy | Otter | Tissue | Included into analysis |  |
| EU573185 | GRA6 | ID 100 | Norway | Fox | Tissue | Included into analysis | [S73] |
| EU573186 | UPRT-intron1 | ID 100 | Norway | Fox | Tissue | Included into analysis |  |
| KC928250 | SAG3 | Pc07 | UK | Polecat | Tissue | Included into analysis | [S77] |
| KC928251 | SAG3 | Pc39 | UK | Polecat | Tissue | Included into analysis |  |
| KC928252 | SAG3 | Pc10 | UK | Polecat | Tissue | Included into analysis |  |
| KC928253 | SAG3 | Pc34 | UK | Polecat | Tissue | Included into analysis |  |
| KC928254 | SAG3 | Ft02 | UK | Ferret | Tissue | Included into analysis |  |
| KC928255 | GRA6 | Pc10 | UK | Polecat | Tissue | Included into analysis |  |
| KC928256 | GRA6 | Pc39 | UK | Polecat | Tissue | Included into analysis |  |
| KC928257 | BTUB | Ft02 | UK | Ferret | Tissue | Included into analysis |  |
| KC928258 | 5'SAG2 | Pc10 | UK | Polecat | Tissue | Included into analysis |  |
| KC928259 | 3'SAG2 | Pc10 | UK | Polecat | Tissue | Included into analysis |  |
| KJ754389 | SAG2 | P1509306 | Portugal | Pigeon | Tissue | Included into analysis | [S52] |
| KJ754390 | SAG2 | P2041924 | Portugal | Pigeon | Tissue | Included into analysis |  |
| KJ754391 | SAG2 | P2307018 | Portugal | Pigeon | Tissue | Included into analysis |  |
| KJ754392 | SAG2 | P2826113 | Portugal | Pigeon | Tissue | Included into analysis |  |
| KJ754393 | SAG2 | P2826320 | Portugal | Pigeon | Tissue | Included into analysis | [S52] |
| KJ754394 | SAG2 | P2996330 | Portugal | Pigeon | Tissue | Included into analysis |  |
| KJ754395 | SAG2 | P2996339 | Portugal | Pigeon | Tissue | Included into analysis |  |
| KJ754396 | SAG2 | P3216020 | Portugal | Pigeon | Tissue | Included into analysis |  |
| KJ754397 | SAG2 | P3439110 | Portugal | Pigeon | Tissue | Included into analysis |  |
| KJ754398 | SAG2 | P3649029 | Portugal | Pigeon | Tissue | Included into analysis |  |
| KJ754399 | SAG2 | P3810413 | Portugal | Pigeon | Tissue | Included into analysis |  |
| KJ754400 | SAG2 | P3884739 | Portugal | Pigeon | Tissue | Included into analysis |  |
| KJ754401 | SAG2 | P4124912 | Portugal | Pigeon | Tissue | Included into analysis |  |
| KJ754402 | SAG2 | P4398917 | Portugal | Pigeon | Tissue | Included into analysis |  |
| KJ754403 | SAG2 | P4510223 | Portugal | Pigeon | Tissue | Included into analysis |  |
| KJ754404 | SAG2 | P4510231 | Portugal | Pigeon | Tissue | Included into analysis |  |
| KJ754405 | SAG2 | P4510237 | Portugal | Pigeon | Tissue | Included into analysis |  |
| KJ754406 | SAG2 | P104405 | Portugal | Pigeon | Tissue | Included into analysis |  |
| KJ754407 | SAG2 | P204828 | Portugal | Pigeon | Tissue | Included into analysis |  |
| KJ754408 | SAG2 | P389538 | Portugal | Pigeon | Tissue | Included into analysis |  |
| KJ754409 | SAG2 | P454205 | Portugal | Pigeon | Tissue | Included into analysis |  |
| KJ754410 | SAG2 | P454210 | Portugal | Pigeon | Tissue | Included into analysis |  |
| KJ754411 | SAG2 | P739933 | Portugal | Pigeon | Tissue | Included into analysis |  |
| KJ754412 | SAG2 | P951505 | Portugal | Pigeon | Tissue | Included into analysis |  |
| KJ754413 | SAG2 | P951520 | Portugal | Pigeon | Tissue | Included into analysis |  |
| KJ754414 | SAG2 | P1076925 | Portugal | Pigeon | Tissue | Included into analysis |  |
| KJ754415 | SAG2 | P1076937 | Portugal | Pigeon | Tissue | Included into analysis |  |
| KJ754416 | SAG2 | P3441710 | Portugal | Pigeon | Tissue | Included into analysis | [S52] |
| KJ754417 | SAG2 | P3441748 | Portugal | Pigeon | Tissue | Included into analysis |  |
| KJ754418 | SAG2 | G2753906 | Portugal | Cat | Tissue | Included into analysis |  |
| KJ754419 | SAG2 | G2808506 | Portugal | Cat | Tissue | Included into analysis |  |
| KJ754420 | SAG2 | G2808513 | Portugal | Cat | Tissue | Included into analysis |  |
| KJ754421 | SAG2 | G2808520 | Portugal | Cat | Tissue | Included into analysis |  |
| KJ754422 | SAG2 | G2863705 | Portugal | Cat | Tissue | Included into analysis |  |
| KJ754423 | SAG2 | G2863709 | Portugal | Cat | Tissue | Included into analysis |  |
| KJ754424 | SAG2 | G2863714 | Portugal | Cat | Tissue | Included into analysis |  |
| KJ754425 | SAG2 | G2992316 | Portugal | Cat | Tissue | Included into analysis |  |
| KJ754426 | SAG2 | G3082702 | Portugal | Cat | Tissue | Included into analysis |  |
| KJ754427 | SAG2 | G3192711 | Portugal | Cat | Tissue | Included into analysis |  |
| KJ754428 | SAG2 | G3861707 | Portugal | Cat | Tissue | Included into analysis |  |
| KJ754429 | SAG2 | G4266412 | Portugal | Cat | Tissue | Included into analysis |  |
| KJ754430 | SAG2 | G101501 | Portugal | Cat | Tissue | Included into analysis |  |
| KJ754431 | SAG2 | G101505 | Portugal | Cat | Tissue | Included into analysis |  |
| KJ754432 | SAG2 | G188404 | Portugal | Cat | Tissue | Included into analysis |  |
| KJ754433 | SAG2 | G188406 | Portugal | Cat | Tissue | Included into analysis |  |
| KJ754434 | SAG2 | G188408 | Portugal | Cat | Tissue | Included into analysis |  |
| KJ754435 | SAG2 | G205601 | Portugal | Cat | Tissue | Included into analysis |  |
| KJ754436 | SAG2 | G205607 | Portugal | Cat | Tissue | Included into analysis |  |
| KJ754437 | SAG2 | G205608 | Portugal | Cat | Tissue | Included into analysis |  |
| KJ754438 | SAG2 | G205619 | Portugal | Cat | Tissue | Included into analysis |  |
| KJ754439 | SAG2 | G1749902 | Portugal | Cat | Tissue | Included into analysis | [S52] |
| KJ754440 | SAG2 | G1749903 | Portugal | Cat | Tissue | Included into analysis |  |
| KJ754441 | SAG2 | G1749904 | Portugal | Cat | Tissue | Included into analysis |  |
| KJ754442 | SAG2 | G2078510 | Portugal | Cat | Tissue | Included into analysis |  |
| KJ754443 | SAG2 | G2078511 | Portugal | Cat | Tissue | Included into analysis |  |
| KJ754444 | SAG2 | G2320002 | Portugal | Cat | Tissue | Included into analysis |  |
| KJ754445 | SAG2 | G2385003 | Portugal | Cat | Tissue | Included into analysis |  |
| KJ754446 | SAG2 | G2385005 | Portugal | Cat | Tissue | Included into analysis |  |
| KJ754447 | SAG2 | G2385012 | Portugal | Cat | Tissue | Included into analysis |  |
| KJ754448 | SAG2 | G2385015 | Portugal | Cat | Tissue | Included into analysis |  |
| KJ754449 | SAG2 | G2385018 | Portugal | Cat | Tissue | Included into analysis |  |
| KJ754450 | SAG2 | G2385019 | Portugal | Cat | Tissue | Included into analysis |  |
| KJ754451 | SAG2 | G2385021 | Portugal | Cat | Tissue | Included into analysis |  |
| KJ754452 | SAG2 | G2385026 | Portugal | Cat | Tissue | Included into analysis |  |
| KJ754453 | SAG2 | G2385028 | Portugal | Cat | Tissue | Included into analysis |  |
| KJ754454 | SAG2 | G2385029 | Portugal | Cat | Tissue | Included into analysis |  |
| KJ754455 | SAG2 | G2385030 | Portugal | Cat | Tissue | Included into analysis |  |
| KJ754456 | SAG2 | G2385031 | Portugal | Cat | Tissue | Included into analysis |  |
| KJ754457 | SAG2 | G2385032 | Portugal | Cat | Tissue | Included into analysis |  |
| KJ754458 | SAG2 | G3441704 | Portugal | Cat | Tissue | Included into analysis |  |
| KJ754459 | SAG2 | G3441705 | Portugal | Cat | Tissue | Included into analysis |  |
| KJ754460 | SAG2 | G3441707 | Portugal | Cat | Tissue | Included into analysis |  |
| KJ754461 | SAG2 | G3441710 | Portugal | Cat | Tissue | Included into analysis |  |
| KJ754462 | SAG2 | G3441801 | Portugal | Cat | Tissue | Included into analysis | [S52] |
| KJ754463 | SAG2 | G3441802 | Portugal | Cat | Tissue | Included into analysis |  |
| KJ754464 | SAG2 | G3441803 | Portugal | Cat | Tissue | Included into analysis |  |
| KJ754465 | SAG2 | G3761302 | Portugal | Cat | Tissue | Included into analysis |  |
| KJ754466 | SAG2 | G3761303 | Portugal | Cat | Tissue | Included into analysis |  |
| KJ754467 | SAG2 | G3761305 | Portugal | Cat | Tissue | Included into analysis |  |
| KJ754468 | SAG2 | G3761306 | Portugal | Cat | Tissue | Included into analysis |  |
| KJ754469 | SAG2 | G3761307 | Portugal | Cat | Tissue | Included into analysis |  |
| KJ754470 | SAG2 | G3761308 | Portugal | Cat | Tissue | Included into analysis |  |
| KJ754471 | SAG2 | G3981301 | Portugal | Cat | Tissue | Included into analysis |  |
| KJ754472 | SAG2 | G3981304 | Portugal | Cat | Tissue | Included into analysis |  |
| KJ754473 | SAG2 | G3981305 | Portugal | Cat | Tissue | Included into analysis |  |
| KJ754474 | SAG2 | G3981310 | Portugal | Cat | Tissue | Included into analysis |  |
| KJ754475 | SAG2 | G4316905 | Portugal | Cat | Tissue | Included into analysis |  |
| KJ754476 | SAG2 | G4316906 | Portugal | Cat | Tissue | Included into analysis |  |
| KJ754477 | SAG2 | G4316907 | Portugal | Cat | Tissue | Included into analysis |  |
| KJ754478 | SAG2 | G4316908 | Portugal | Cat | Tissue | Included into analysis |  |
| KJ754479 | SAG2 | G4316911 | Portugal | Cat | Tissue | Included into analysis |  |
| KJ754480 | SAG2 | G5119505 | Portugal | Cat | Tissue | Included into analysis |  |
| KJ754481 | SAG2 | G1367306 | Portugal | Cat | Tissue | Included into analysis |  |
| KJ754482 | SAG2 | G1367309 | Portugal | Cat | Tissue | Included into analysis |  |
| KJ754483 | SAG2 | G1367410 | Portugal | Cat | Tissue | Included into analysis |  |
| KJ754484 | SAG2 | G1367412 | Portugal | Cat | Tissue | Included into analysis |  |
| KJ754485 | SAG2 | G1367414 | Portugal | Cat | Tissue | Included into analysis | [S52] |
| KJ754486 | SAG2 | G1367502 | Portugal | Cat | Tissue | Included into analysis |  |
| KJ754487 | SAG2 | G1367503 | Portugal | Cat | Tissue | Included into analysis |  |
| KJ754488 | SAG2 | G1367505 | Portugal | Cat | Tissue | Included into analysis |  |
| KJ754489 | SAG2 | G1367509 | Portugal | Cat | Tissue | Included into analysis |  |
| KJ754490 | SAG2 | G1367405 | Portugal | Cat | Tissue | Included into analysis |  |
| KJ754491 | SAG2 | G5119511 | Portugal | Cat | Tissue | Included into analysis |  |
| KJ754492 | SAG2 | G5119601 | Portugal | Cat | Tissue | Included into analysis |  |
| KJ754493 | SAG2 | G3192710 | Portugal | Cat | Tissue | Included into analysis |  |
| KJ754494 | SAG2 | G188409 | Portugal | Cat | Tissue | Included into analysis |  |
| KJ754495 | SAG2 | G3981308 | Portugal | Cat | Tissue | Included into analysis |  |
| KJ754496 | SAG2 | G4316912 | Portugal | Cat | Tissue | Included into analysis |  |
| KJ754497 | SAG2 | G1367411 | Portugal | Cat | Tissue | Included into analysis |  |
| KJ754498 | SAG2 | G2385033 | Portugal | Cat | Tissue | Included into analysis |  |
| KJ754499 | SAG2 | G2385034 | Portugal | Cat | Tissue | Included into analysis |  |
| JX218225 | SAG3 | RH | Reference | Type | I | References included to analysis | - |
| SAG3_ME49_trunc_ToxoDB47 | SAG3 | ME49 | Reference | Type | II | References included to analysis | - |
| SAG3_VEG_trunc_ToxoDB47 | SAG3 | VEG | Reference | Type | III | References included to analysis | - |
| MT321285 | GRA6 | ME49 | Reference | Type | II | References included to analysis | - |
| AF239285 | GRA6 | ME49 | Reference | Type | II | References included to analysis | - |
| AF239283 | GRA6 | RH | Reference | Type | I | References included to analysis | - |
| JX044209 | GRA6 | VEG | Reference | Type | III | References included to analysis | - |
| MN275918 | B1 | RH | Reference | Type | I | References included to analysis | - |
| MN275919 | B1 | PRU | Reference | Type | II | References included to analysis | - |
| MN275920 | B1 | VEG | Reference | Type | III | References included to analysis | - |

**References**

1. Petersen, E. *et al*. (2006) Diagnosis of pulmonary infection with *Toxoplasma gondii* in immunocompromised HIV-positive patients by real-time PCR. *Eur. J. Clin. Microbiol. Infect. Dis.* 25, 401-404
2. Costa, J.M. *et al*. (2013) Direct genotyping of *Toxoplasma gondii* from amniotic fluids based on B1 gene polymorphism using minisequencing analysis. *BMC Infect. Dis.* 13, 552-2334-13-552
3. Edvinsson, B. *et al*. (2007) Rapid genotyping of *Toxoplasma gondii* by pyrosequencing. *Clin. Microbiol. Infect.* 13, 424-429
4. Howe, D.K. *et al*. (1997) Determination of genotypes of *Toxoplasma gondii* strains isolated from patients with toxoplasmosis. *J. Clin. Microbiol.* 35, 1411-1414
5. Haque, S. *et al*. (1999) Infection of mice by a *Toxoplasma gondii* isolate from an AIDS patient: virulence and activation of hosts' immune responses are independent of parasite genotype. *Parasite Immunol.* 21, 649-657
6. Honore, S. *et al*. (2000) Genotyping of *Toxoplasma gondii* strains from immunocompromised patients. *Pathol. Biol. (Paris)* 48, 541-547
7. Herrmann, D.C. *et al*. (2014) Genotyping of samples from German patients with ocular, cerebral and systemic toxoplasmosis reveals a predominance of *Toxoplasma gondii* type II. *Int. J. Med. Microbiol.* 304, 911-916
8. Messaritakis, I. *et al*. (2008) Prevalent genotypes of *Toxoplasma gondii* in pregnant women and patients from Crete and Cyprus. *Am. J. Trop. Med. Hyg.* 79, 205-209
9. Antoniou, M. *et al*. (2007) Toxoplasmosis in pregnant women in Crete. *Parassitologia* 49, 231-233
10. Pagliuca, C. *et al*. (2017) Genotyping of *Toxoplasma gondii* strain directly from human CSF samples of congenital toxoplasmosis clinical case. *New Microbiol.* 40, 151-154
11. Nowakowska, D. *et al*. (2006) Genotyping of *Toxoplasma gondii* by multiplex PCR and peptide-based serological testing of samples from infants in Poland diagnosed with congenital toxoplasmosis. *J. Clin. Microbiol.* 44, 1382-1389
12. Switaj, K. *et al*. (2006) Association of ocular toxoplasmosis with type I *Toxoplasma gondii* strains: direct genotyping from peripheral blood samples. *J. Clin. Microbiol.* 44, 4262-4264
13. Vilares, A. *et al*. (2017) Molecular and virulence characterization of *Toxoplasma gondii* strains isolated from humans in Portugal. *Parasitol. Res.* 116, 979-985
14. Djurkovic-Djakovic, O. *et al*. (2006) A human origin type II strain of *Toxoplasma gondii* causing severe encephalitis in mice. *Microbes Infect.* 8, 2206-2212
15. Markovic, M. *et al*. (2014) Evidence for genetic diversity of *Toxoplasma gondii* in selected intermediate hosts in Serbia. *Comp. Immunol. Microbiol. Infect. Dis.* 37, 173-179
16. Stajner, T. *et al*. (2013) Atypical strain of *Toxoplasma gondii* causing fatal reactivation after hematopoietic stem cell transplantion in a patient with an underlying immunological deficiency. *J. Clin. Microbiol.* 51, 2686-2690
17. Turcekova, L. *et al*. (2012) Molecular diagnosis of *Toxoplasma gondii* in pregnant women. *Bratisl. Lek. Listy* 113, 307-310
18. Fuentes, I. *et al*. (2001) Genotypic characterization of *Toxoplasma gondii* strains associated with human toxoplasmosis in Spain: direct analysis from clinical samples. *J. Clin. Microbiol.* 39, 1566-1570
19. Burrells, A. *et al*. (2016) The prevalence and genotypic analysis of *Toxoplasma gondii* from individuals in Scotland, 2006-2012. *Parasit. Vectors* 9, 324-016-1610-6
20. Aspinall, T.V. *et al*. (2003) Molecular evidence for multiple *Toxoplasma gondii* infections in individual patients in England and Wales: public health implications. *Int. J. Parasitol.* 33, 97-103
21. Mancianti, F. *et al*. (2014) Detection and genotyping of *Toxoplasma gondii* DNA in the blood and milk of naturally infected donkeys (*Equus asinus*). *Parasit. Vectors* 7, 165-3305-7-165
22. Papini, R.A. *et al*. (2015) Seroprevalence and Genotyping *of Toxoplasma gondii* in Horses Slaughtered for Human Consumption in Italy. *Journal of Equine Veterinary Science* 35, 657-661
23. Mancianti, F. *et al*. (2013) Seroprevalence, detection of DNA in blood and milk, and genotyping of *Toxoplasma gondii* in a goat population in Italy. *Biomed. Res. Int.* 2013, 905326
24. Gazzonis, A.L. *et al*. (2020) *Toxoplasma gondii* infection in meat-producing small ruminants: Meat juice serology and genotyping. *Parasitol. Int.* 76, 102060
25. Pavone, S. *et al*. (2020) Fatal systemic toxoplasmosis in a 3-month-old young tibetan goat (*Capra hircus*). *BMC Vet. Res.* 16, 423-020-02641-8
26. Sroka, J. *et al*. (2017) Seroprevalence of *Toxoplasma gondii* infection in goats from the south-west region of Poland and the detection of T. gondii DNA in goat milk. *Folia. Parasitol. (Praha)* 64, 10.14411/fp.2017.023
27. Lopes, A.P. *et al*. (2015) Genotyping Characterization of *Toxoplasma gondii* in Cattle, Sheep, Goats and Swine from the North of Portugal. *Iran. J. Parasitol.* 10, 465-472
28. Spišák, F. *et al*. (2010) Prevalence estimation and genotypization of *Toxoplasma gondii* in goats. *Biologia* 65, 670-674
29. Battisti, E. *et al*. (2018) Circulating genotypes of *Toxoplasma gondii* in Northwestern Italy. *Vet. Parasitol.* 253, 43-47
30. Verma, S. *et al*. (2015) Genetic characterization of *Toxoplasma gondii* isolates from Portugal, Austria and Israel reveals higher genetic variability within the type II lineage. *Parasitology* 142, 948-957
31. Berger-Schoch, A.E. *et al*. (2011) Prevalence and genotypes of *Toxoplasma gondii* in feline faeces (oocysts) and meat from sheep, cattle and pigs in Switzerland. *Vet. Parasitol.* 177, 290-297
32. Aspinall, T.V. *et al*. (2002) Prevalence of *Toxoplasma gondii* in commercial meat products as monitored by polymerase chain reaction--food for thought? *Int. J. Parasitol.* 32, 1193-1199
33. Slany, M. *et al*. (2016) Molecular characterization of *Toxoplasma gondii* in pork meat from different production systems in the Czech Republic. *Int. J. Food Microbiol.* 238, 252-255
34. Djokic, V. *et al*. (2016) *Toxoplasma gondii* infection in pork produced in France. *Parasitology* 143, 557-567
35. Papini, R. *et al*. (2017) Occurrence of *Toxoplasma gondii* in Carcasses of Pigs Reared in Intensive Systems in Northern Italy. *J. Food Prot.* 80, 515-522
36. Gazzonis, A.L. *et al*. (2018) *Toxoplasma gondii* infection and biosecurity levels in fattening pigs and sows: serological and molecular epidemiology in the intensive pig industry (Lombardy, Northern Italy). *Parasitol. Res.* 117, 539-546
37. Vergara, A. *et al*. (2018) *Toxoplasma gondii* Lineages Circulating in Slaughtered Industrial Pigs and Potential Risk for Consumers. *J. Food Prot.* 81, 1373-1378
38. Sroka, J. *et al*. (2020) *Toxoplasma gondii* infection in slaughtered pigs and cattle in Poland: seroprevalence, molecular detection and characterization of parasites in meat. *Parasit. Vectors* 13, 223-020-04106-1
39. de Sousa, S. *et al*. (2006) Biologic and molecular characterization of *Toxoplasma gondii* isolates from pigs from Portugal. *Vet. Parasitol.* 135, 133-136
40. Kuruca, L. *et al*. (2019) *Toxoplasma gondii* genotypes circulating in domestic pigs in Serbia. *Acta Vet. Hung.* 67, 204-211
41. Turcekova, L. *et al*. (2013) Occurrence and genetic characterization of *Toxoplasma gondii* in naturally infected pigs. *Acta Parasitol.* 58, 361-366
42. Fernandez-Escobar, M. *et al*. (2020) Isolation, Genotyping, and Mouse Virulence Characterization of *Toxoplasma gondii* From Free Ranging Iberian Pigs. *Front. Vet. Sci.* 7, 604782
43. Halos, L. *et al*. (2010) An innovative survey underlining the significant level of contamination by *Toxoplasma gondii* of ovine meat consumed in France. *Int. J. Parasitol.* 40, 193-200
44. Gutierrez, J. *et al*. (2012) Application of quantitative real-time polymerase chain reaction for the diagnosis of toxoplasmosis and enzootic abortion of ewes. *J. Vet. Diagn. Invest.* 24, 846-854
45. Vismarra, A. *et al*. (2017) *Toxoplasma gondii* and Pre-treatment Protocols for Polymerase Chain Reaction Analysis of Milk Samples: A Field Trial in Sheep from Southern Italy. *Ital. J. Food Saf.* 6, 6501
46. Vismarra, A. *et al*. (2017) *Toxoplasma gondii* in the Cornigliese sheep breed in Italy: Meat juice serology, in vitro isolation and genotyping. *Vet. Parasitol.* 243, 125-129
47. Chessa, G. *et al*. (2014) Molecular characterization of *Toxoplasma gondii* Type II in sheep abortion in Sardinia, Italy. *Parasite* 21, 6
48. Fernandez-Escobar, M. *et al*. (2020) Isolation and genetic characterization of *Toxoplasma gondii* in Spanish sheep flocks. *Parasit. Vectors* 13, 396-020-04275-z
49. Opsteegh, M. *et al*. (2010) Direct detection and genotyping of *Toxoplasma gondii* in meat samples using magnetic capture and PCR. *Int. J. Food Microbiol.* 139, 193-201
50. Owen, M. and Trees, A. (1999) Genotyping of *Toxoplasma gondii* associated with abortion in sheep. *J. Parasitol.* 382-384
51. Mancianti, F. *et al*. (2015) A retrospective molecular study of select intestinal protozoa in healthy pet cats from Italy. *J. Feline Med. Surg.* 17, 163-167
52. Vilares, A. *et al*. (2014) Isolation and molecular characterization of *Toxoplasma gondii* isolated from pigeons and stray cats in Lisbon, Portugal. *Vet. Parasitol.* 205, 506-511
53. Montoya, A. *et al*. (2008) Molecular characterization of *Toxoplasma gondii* isolates from cats in Spain. *J. Parasitol.* 94, 1044-1046
54. Spycher, A. *et al*. (2011) Isolation and genotyping of *Toxoplasma gondii* causing fatal systemic toxoplasmosis in an immunocompetent 10-year-old cat. *J. Vet. Diagn. Invest.* 23, 104-108
55. Herrmann, D.C. *et al*. (2010) Atypical *Toxoplasma gondii* genotypes identified in oocysts shed by cats in Germany. *Int. J. Parasitol.* 40, 285-292
56. Schares, G. *et al*. (2008) Occurrence of *Toxoplasma gondii* and *Hammondia hammondi* oocysts in the faeces of cats from Germany and other European countries. *Vet. Parasitol.* 152, 34-45
57. Migliore, S. *et al*. (2017) A rare case of acute toxoplasmosis in a stray dog due to infection of *T. gondii* clonal type I: public health concern in urban settings with stray animals? *BMC Vet. Res.* 13, 249-017-1176-3
58. Dubey, J.P. *et al*. (2008) Seroprevalence and isolation of *Toxoplasma gondii* from free-range chickens in Ghana, Indonesia, Italy, Poland, and Vietnam. *J. Parasitol.* 94, 68-71
59. Schares, G. *et al*. (2017) High seroprevalence of *Toxoplasma gondii* and probability of detecting tissue cysts in backyard laying hens compared with hens from large free-range farms. *Int. J. Parasitol.* 47, 765-777
60. Dubey, J.P. *et al*. (2005) Genetic and biologic characteristics of *Toxoplasma gondii* infections in free-range chickens from Austria. *Vet. Parasitol.* 133, 299-306
61. Dubey, J.P. *et al*. (2006) Characterization of *Toxoplasma gondii* isolates in free-range chickens from Portugal. *J. Parasitol.* 92, 184-186
62. Bajnok, J. *et al*. (2015) Prevalence of Toxoplasma gondii in localized populations of *Apodemus sylvaticus* is linked to population genotype not to population location. *Parasitology* 142, 680-690
63. Herrmann, D.C. *et al*. (2013) Genetic characterisation of *Toxoplasma gondii* isolates from European beavers (*Castor fiber*) and European wildcats (*Felis silvestris silvestris*). *Vet. Parasitol.* 191, 108-111
64. Fernández-Escobar, M. *et al*. (2020) Molecular survey for cyst-forming coccidia (*Toxoplasma gondii*, *Neospora caninum*, *Sarcocystis* spp.) in Mediterranean periurban micromammals. *Parasitol. Res.* 119, 2679-2686
65. Ivovic, V. *et al*. (2019) Prevalence and genotype identification of *Toxoplasma gondii* in suburban rodents collected at waste disposal sites. *Parasite* 26, 27
66. Sroka, J. *et al*. (2019) *Toxoplasma gondii* infection in selected species of free-living animals in Poland. *Ann. Agric. Environ. Med.* 26, 656-660
67. Turcekova, L. *et al*. (2014) *Toxoplasma gondii* in protected wildlife in the Tatra National Park (TANAP), Slovakia. *Ann. Agric. Environ. Med.* 21, 235-238
68. Di Guardo, G. *et al*. (2011) Genotyping of *Toxoplasma gondii* isolates in meningo-encephalitis affected striped dolphins (*Stenella coeruleoalba*) from Italy. *Vet. Parasitol.* 183, 31-36
69. Terracciano, G. *et al*. (2020) Dolphins Stranded along the Tuscan Coastline (Central Italy) of the "Pelagos Sanctuary": A Parasitological Investigation. *Pathogens* 9, 10.3390/pathogens9080612
70. Giorda, F. *et al*. (2021) Evidence for Unknown *Sarcocystis*-Like Infection in Stranded Striped Dolphins (*Stenella coeruleoalba*) from the Ligurian Sea, Italy. *Animals (Basel)* 11, 10.3390/ani11051201
71. Herder, V. *et al*. (2015) Fatal Disseminated *Toxoplasma gondii* Infection in a Captive Harbour Porpoise (*Phocoena phocoena*). *J. Comp. Pathol.* 153, 357-362
72. Prestrud, K.W. *et al*. (2008) First isolate of *Toxoplasma gondii* from arctic fox (*Vulpes lagopus*) from Svalbard. *Vet. Parasitol.* 151, 110-114
73. Prestrud, K.W. *et al*. (2008) Direct high-resolution genotyping of *Toxoplasma gondii* in arctic foxes (*Vulpes lagopus*) in the remote arctic Svalbard archipelago reveals widespread clonal Type II lineage. *Vet. Parasitol.* 158, 121-128
74. Herrmann, D.C. *et al*. (2012) *Toxoplasma gondii* in foxes and rodents from the German Federal States of Brandenburg and Saxony-Anhalt: seroprevalence and genotypes. *Vet. Parasitol.* 185, 78-85
75. Verin, R. *et al*. (2013) Serologic, molecular, and pathologic survey of *Toxoplasma gondii* infection in free-ranging red foxes (*Vulpes vulpes*) in central Italy. *J. Wildl. Dis.* 49, 545-551
76. Uzelac, A. *et al*. (2019) Detection and genotyping of *Toxoplasma gondii* in wild canids in Serbia. *Parasitol. Int.* 73, 101973
77. Burrells, A. *et al*. (2013) Evidence of the three main clonal *Toxoplasma gondii* lineages from wild mammalian carnivores in the UK. *Parasitology* 140, 1768-1776
78. Moskwa, B. *et al*. (2017) First *Toxoplasma gondii* isolate from an aborted foetus of European bison (*Bison bonasus bonasus* L.). *Parasitol. Res.* 116, 2457-2461
79. Formenti, N. *et al*. (2016) Spread and genotype of *Toxoplasma gondii* in naturally infected alpine chamois (*Rupicapra r. rupicapra*). *Parasitol. Res.* 115, 2115-2120
80. Plaza, J. *et al*. (2020) Detection of *Toxoplasma gondii* in retail meat samples in Scotland. *Food Waterborne Parasitol.* 20, e00086
81. Rocchigiani, G. *et al*. (2016) Seroprevalence of *Toxoplasma gondii* and *Neospora caninum* in red deer from Central Italy. *Ann. Agric. Environ. Med.* 23, 699-701
82. Aubert, D. *et al*. (2010) Molecular and biological characteristics of *Toxoplasma gondii* isolates from wildlife in France. *Vet. Parasitol.* 171, 346-349
83. Calero-Bernal, R. *et al*. (2015) Prevalence and genotype identification of *Toxoplasma gondii* in wild animals from Southwestern Spain. *J. Wildl. Dis.* 51, 233-238
84. Richomme, C. *et al*. (2009) Genetic characterization of *Toxoplasma gondii* from wild boar (*Sus scrofa*) in France. *Vet. Parasitol.* 164, 296-300
85. Bacci, C. *et al*. (2015) Detection of *Toxoplasma gondii* in free-range, organic pigs in Italy using serological and molecular methods. *Int. J. Food Microbiol.* 202, 54-56
86. Gazzonis, A.L. *et al*. (2018) Occurrence of selected zoonotic food-borne parasites and first molecular identification of *Alaria alata* in wild boars (*Sus scrofa*) in Italy. *Parasitol. Res.* 117, 2207-2215
87. Calero-Bernal, R. *et al*. (2013) Congenital toxoplasmosis in wild boar (*Sus scrofa*) and identification of the *Toxoplasma gondii* types involved. *J. Wildl. Dis.* 49, 1019-1023
88. Mancianti, F. *et al*. (2020) Epidemiologic Survey on *Toxoplasma gondii* and *Trichinella pseudospiralis* Infection in Corvids from Central Italy. *Pathogens* 9, 10.3390/pathogens9050336
89. Mancianti, F. *et al*. (2013) *Toxoplasma gondii* in waterfowl: the first detection of this parasite in *Anas crecca* and *Anas clypeata* from Italy. *J. Parasitol.* 99, 561-563
90. Skorpikova, L. *et al*. (2018) Molecular detection of *Toxoplasma gondii* in feathered game intended for human consumption in the Czech Republic. *Int. J. Food Microbiol.* 286, 75-79
91. Slany, M. *et al*. (2019) *Toxoplasma gondii* in vegetables from fields and farm storage facilities in the Czech Republic. *FEMS Microbiol. Lett.* 366, 10.1093/femsle/fnz170
92. Lass, A. *et al*. (2012) The first detection of *Toxoplasma gondii* DNA in environmental fruits and vegetables samples. *Eur. J. Clin. Microbiol. Infect. Dis.* 31, 1101-1108
93. Caradonna, T. *et al*. (2017) Detection and prevalence of protozoan parasites in ready-to-eat packaged salads on sale in Italy. *Food Microbiol.* 67, 67-75
94. Lass, A. *et al*. (2009) Detection of *Toxoplasma gondii* oocysts in environmental soil samples using molecular methods. *Eur. J. Clin. Microbiol. Infect. Dis.* 28, 599-605
95. Adamska, M. (2018) Molecular detection of *Toxoplasma gondii* in natural surface water bodies in Poland. *J. Water. Health.* 16, 657-660
96. Sroka, J. *et al*. (2010) The occurrence of *Toxoplasma gondii* infection in people and animals from rural environment of Lublin region - estimate of potential role of water as a source of infection. *Ann. Agric. Environ. Med.* 17, 125-132
97. Lass, A. *et al*. (2017) The first detection of *Toxoplasma gondii* DNA in environmental air samples using gelatine filters, real-time PCR and loop-mediated isothermal (LAMP) assays: qualitative and quantitative analysis. *Parasitology* 144, 1791-1801
98. Wojcik-Fatla, A. *et al*. (2015) *Toxoplasma gondii* (Nicolle et Manceaux, 1908) detected in *Dermacentor reticulatus* (Fabricius) (Ixodidae). *Folia. Parasitol. (Praha)* 62, 10.14411/fp.2015.055
99. Adamska, M. and Skotarczak, B. (2017) Molecular evidence for *Toxoplasma gondii* in feeding and questing *Ixodes ricinus* ticks. *Ticks Tick Borne Dis.* 8, 259-261
100. Sroka, J. *et al*. (2008) Preliminary study on the occurrence of *Toxoplasma gondii* in *Ixodes ricinus* ticks from north-western Poland with the use of PCR. *Ann. Agric. Environ. Med.* 15, 333-338
101. Sroka, J. *et al*. (2009) The occurrence of *Toxoplasma gondii* and *Borrelia burgdorferi* sensu lato in *Ixodes ricinus* ticks from east Poland with the use of pcr. *Ann. Agric. Environ. Med.* 16, 313-319
102. Ajzenberg, D. *et al*. (2009) Genotype of 88 *Toxoplasma gondii* isolates associated with toxoplasmosis in immunocompromised patients and correlation with clinical findings. *J. Infect. Dis.* 199, 1155-1167
103. Ajzenberg, D. *et al*. (2010) Genotyping of *Toxoplasma gondii* isolates with 15 microsatellite markers in a single multiplex PCR assay. *J. Clin. Microbiol.* 48, 4641-4645
104. Gisbert Algaba, I. *et al*. (2020) Molecular Study of *Toxoplasma gondii* Isolates Originating from Humans and Organic Pigs in Belgium. *Foodborne Pathog. Dis.* 17, 316-321
105. Shwab, E.K. *et al*. (2018) Human impact on the diversity and virulence of the ubiquitous zoonotic parasite *Toxoplasma gondii*. *Proc. Natl. Acad. Sci. U. S. A.* 115, E6956-E6963
106. Jokelainen, P. *et al*. (2018) Direct genetic characterization of *Toxoplasma gondii* from clinical samples from Denmark: not only genotypes II and III. *Eur. J. Clin. Microbiol. Infect. Dis.* 37, 579-586
107. Mercier, A. *et al*. (2011) Human impact on genetic diversity of *Toxoplasma gondii*: example of the anthropized environment from French Guiana. *Infect. Genet. Evol.* 11, 1378-1387
108. Ajzenberg, D. *et al*. (2004) Genetic diversity, clonality and sexuality in *Toxoplasma gondii*. *Int. J. Parasitol.* 34, 1185-1196
109. Ajzenberg, D. *et al*. (2015) The rural-urban effect on spatial genetic structure of type II *Toxoplasma gondii* strains involved in human congenital toxoplasmosis, France, 2002-2009. *Infect. Genet. Evol.* 36, 511-516
110. Costache, C.A. *et al*. (2013) First isolation and genetic characterization of a *Toxoplasma gondii* strain from a symptomatic human case of congenital toxoplasmosis in Romania. *Parasite* 20, 11
111. De Salvador-Guillouet, F. *et al*. (2006) Severe pneumonia during primary infection with an atypical strain of *Toxoplasma gondii* in an immunocompetent young man. *J. Infect.* 53, e47-50
112. Delhaes, L. *et al*. (2010) Severe congenital toxoplasmosis due to a *Toxoplasma gondii* strain with an atypical genotype: case report and review. *Prenat. Diagn.* 30, 902-905
113. Fekkar, A. *et al*. (2011) Direct genotyping of *Toxoplasma gondii* in ocular fluid samples from 20 patients with ocular toxoplasmosis: predominance of type II in France. *J. Clin. Microbiol.* 49, 1513-1517
114. Ghosn, J. *et al*. (2003) Atypical toxoplasmic manifestation after discontinuation of maintenance therapy in a human immunodeficiency virus type 1-infected patient with immune recovery. *Clin. Infect. Dis.* 37, e112-4
115. Khan, A. *et al*. (2014) Geographic separation of domestic and wild strains of *Toxoplasma gondii* in French Guiana correlates with a monomorphic version of chromosome1a. *PLoS Negl Trop. Dis.* 8, e3182
116. Klun, I. *et al*. (2017) The first isolation and molecular characterization of *Toxoplasma gondii* from horses in Serbia. *Parasit. Vectors* 10, 167-017-2104-x
117. Martinot, M. *et al*. (2020) Spinal cord toxoplasmosis in a young immunocompetent patient. *Infection* 48, 299-302
118. Su, C. *et al*. (2012) Globally diverse *Toxoplasma gondii* isolates comprise six major clades originating from a small number of distinct ancestral lineages. *Proc. Natl. Acad. Sci. U. S. A.* 109, 5844-5849
119. Genot, S. *et al*. (2007) Severe *Toxoplasma gondii* I/III recombinant-genotype encephalitis in a human immunodeficiency virus patient. *J. Clin. Microbiol.* 45, 3138-3140
120. Blaga, R. *et al*. (2019) *Toxoplasma gondii* in beef consumed in France: regional variation in seroprevalence and parasite isolation. *Parasite* 26, 77
121. Pastiu, A.I. *et al*. (2019) Prevalence and genetic characterization of *Toxoplasma gondii* in naturally infected backyard pigs intended for familial consumption in Romania. *Parasit. Vectors* 12, 586-019-3842-8
122. Bertranpetit, E. *et al*. (2017) Phylogeography of *Toxoplasma gondii* points to a South American origin. *Infection, Genetics and Evolution* 48, 150-155
123. Dumètre, A. *et al*. (2006) *Toxoplasma gondii* infection in sheep from Haute-Vienne, France: seroprevalence and isolate genotyping by microsatellite analysis. *Vet. Parasitol.* 142, 376-379
124. Jokelainen, P. *et al*. (2012) Feline toxoplasmosis in Finland: cross-sectional epidemiological study and case series study. *J. Vet. Diagn. Invest.* 24, 1115-1124
125. Marcer, F. *et al*. (2019) Parasitological and pathological findings in fin whales *Balaenoptera physalus* stranded along Italian coastlines. *Dis. Aquat. Organ.* 133, 25-37
126. Sgroi, G. *et al*. (2020) Genotyping of *Toxoplasma gondii* in wild boar (*Sus scrofa*) in southern Italy: Epidemiological survey and associated risk for consumers. *Zoonoses Public. Health.* 67, 805-813
127. Gamble, A. *et al*. (2019) Exposure of yellow-legged gulls to *Toxoplasma gondii* along the Western Mediterranean coasts: Tales from a sentinel. *Int. J. Parasitol. Parasites Wildl.* 8, 221-228
128. De Craeye, S. *et al*. (2011) *Toxoplasma gondii* and *Neospora caninum* in wildlife: common parasites in Belgian foxes and Cervidae? *Vet. Parasitol.* 178, 64-69
129. Lukasova, R. *et al*. (2018) Molecular Evidence of *Toxoplasma gondii*, *Neospora caninum*, and *Encephalitozoon cuniculi* in Red Foxes (*Vulpes vulpes*). *J. Wildl. Dis.* 54, 825-828
130. Racka, K. *et al*. (2020) Fatal toxoplasmosis in wild European brown hares (*Lepus europaeus*) in tularaemia endemic areas of the Czech Republic: Poses risk of infection for humans? *Transbound Emerg. Dis.* *doi: 10.1111/tbed.13925.*
131. Jokelainen, P. *et al*. (2011) Natural *Toxoplasma gondii* infections in European brown hares and mountain hares in Finland: proportional mortality rate, antibody prevalence, and genetic characterization. *J. Wildl. Dis.* 47, 154-163
132. Jokelainen, P. and Nylund, M. (2012) Acute fatal toxoplasmosis in three Eurasian red squirrels (*Sciurus vulgaris*) caused by genotype II of *Toxoplasma gondii*. *J. Wildl. Dis.* 48, 454-457
133. Machačová, T. *et al*. (2016) *Toxoplasma gondii* and *Neospora caninum* in wild small mammals: seroprevalence, DNA detection and genotyping. *Vet. Parasitol.* 223, 88-90
134. Santoro, M. *et al*. (2020) Parasite Load and STRs Genotyping of *Toxoplasma gondii* Isolates From Mediterranean Mussels (*Mytilus galloprovincialis*) in Southern Italy. *Front. Microbiol.* 11, 355
135. Viscardi, M. *et al*. (2021) A type II variant of *Toxoplasma gondii* infects the Eurasian otter (*Lutra lutra*) in southern Italy. *Transbound Emerg. Dis. doi: 10.1111/tbed.14012.*
136. Sroka, J. *et al*. (2019) Detection and Molecular Characteristics of *Toxoplasma gondii* DNA in Retail Raw Meat Products in Poland. *Foodborne Pathog. Dis.* 16, 195-204
137. Sousa, S. *et al*. (2009) Selection of polymorphic peptides from GRA6 and GRA7 sequences of *Toxoplasma gondii* strains to be used in serotyping. *Clin. Vaccine Immunol.* 16, 1158-1169
138. Azimpour-Ardakan, T. *et al*. (2021) Designing and developing of high-resolution melting technique for separating different types of *Toxoplasma gondii* by analysis of B1 and ROP8 gene regions. *J. Microbiol. Methods* 184, 106188
139. Tedde, T. *et al*. (2019) Toxoplasma gondii and Other Zoonotic Protozoans in Mediterranean Mussel (*Mytilus galloprovincialis*) and Blue Mussel (*Mytilus edulis*): A Food Safety Concern? *J. Food Prot.* 82, 535-542
140. Vichova, B. *et al*. (2016) Molecular screening for bacteria and protozoa in great cormorants (*Phalacrocorax carbo sinensis*) nesting in Slovakia, central Europe. *Acta Parasitol.* 61, 585-589
